# Metformin Ameliorates Hepatic Steatosis induced by olanzapine through inhibiting LXRα/PCSK9 pathway

**DOI:** 10.1038/s41598-022-09610-1

**Published:** 2022-04-04

**Authors:** Wenqiang Zhu, Chen Ding, Piaopiao Huang, Juanli Ran, Pingan Lian, Yaxin Tang, Wen Dai, Xiansheng Huang

**Affiliations:** 1 Department of Cardiovascular Medicine, The Second Xiangya Hospital, Central South University, Changsha, 410011 China; 2 Department of Stomatology, The Second Xiangya Hospital, Central South University, Changsha, 410011 China; 3 Department of Medicine, Columbia University Medical Center, New York, USA

**Keywords:** Diseases, Medical research

## Abstract

Studies have confirmed that olanzapine, the mainstay treatment for schizophrenia, triggers metabolic diseases, including non-alcoholic fatty liver disease (NAFLD). However, the etiology of olanzapine-induced NAFLD is poorly understood. Proprotein convertase subtilisin kexin type 9 (PCSK9) is involved in NAFLD pathogenesis, and metformin can significantly decrease circulating PCSK9. The purpose of this study was to investigate the role of PCSK9 and explore the therapeutic effect of metformin for olanzapine-associated NAFLD. Olanzapine significantly upregulated PCSK9 and promoted lipid accumulation in mouse livers and HepG2 and AML12 cells. Metformin ameliorated these pathological alterations. PCSK9 upstream regulator liver X receptor α (LXRα) was significantly upregulated in olanzapine-induced NAFLD. LXRα antagonist treatment and LXRα overexpression resulted in a decrease and increase of PCSK9, respectively. Hepatic lipogenesis-associated genes FAS and SCD1 were significantly upregulated in olanzapine-induced NAFLD mice and HepG2 cells overexpressing PCSK9, and genes related to lipid β-oxidation (SCAD and PPARα) were downregulated, while metformin reversed these changes. In addition, we found that LXRα overexpression compromised the effect of metformin on PCSK9 levels and intracellular lipid droplet formation. Taken together, our findings suggest that olanzapine enhances hepatic PCSK9 expression by upregulating LXRα, thereby increasing FAS and SCD1 expression as well as decreasing SCAD and PPARα, and promoting lipid accumulation, and, subsequently, NAFLD, which is ameliorated by metformin.

## Introduction

Non-alcoholic fatty liver disease (NAFLD), a fatty liver disease which develops in the absence of excessive alcohol consumption, is the most prevalent hepatic condition worldwide^1^. NAFLD begins with the aberrant accumulation of triglycerides within the liver and progresses from simple hepatic steatosis to non-alcoholic steatohepatitis, hepatic cirrhosis, and even hepatocellular carcinoma^1^. Moreover, NAFLD contributes to cardiovascular disease, which in turn is a major cause of mortality in patients with NAFLD^2^. This hepatic condition is strongly associated with obesity, insulin resistance, type 2 diabetes, and dyslipidemia. Remarkably, long-term treatment with olanzapine, one of the most used drugs for schizophrenia, dramatically increases the risk of obesity and metabolic dysregulation^3^. Therefore, olanzapine treatment confers susceptibility to NAFLD, as confirmed by previous preclinical studies and in vitro models^4,5^, including our own research. However, the pathogenesis of olanzapine-induced NAFLD remains poorly understood, and no optimal therapeutics have been established.

Metformin, the first-line pharmacologic treatment and the most commonly prescribed drug for type 2 diabetes, improves dyslipidemia, insulin resistance, and obesity^6^. The pleiotropic effects of metformin include a lower risk of cardiovascular disease and, possibly, NAFLD. In contrast, most traditional agents for hyperglycemia, including insulin^7^, sulfonylureas, and glucokinase activators^8^ cannot effectively decrease the risk of major cardiovascular events due to their limited effects on dyslipidemia and obesity despite glucose control. Animal and human studies have shown that metformin acts in the liver, where it inhibits gluconeogenesis and lipogenesis, thus attenuating the development of NAFLD. Indeed, the efficacy of metformin for NAFLD has been established in previous studies by our group^9^ and others^10^. Interestingly, metformin has been shown to alleviate olanzapine-induced weight gain, insulin resistance, and dyslipidemia^9^. Thus, metformin could theoretically ameliorate olanzapine-induced NAFLD.

Recently, proprotein convertase subtilisin kexin type 9 (PCSK9) has been implicated in the pathogenesis of NAFLD^11^. PCSK9 promotes the degradation of the low-density lipoprotein (LDL) receptor (LDLR), thus reducing hepatocyte surface LDLR expression, which results in higher LDL cholesterol (LDL-C) plasma levels and, subsequently, a higher risk of cardiovascular disease^11^. Gain-of-function mutations in the PCSK9 gene predispose patients to cardiovascular disease^12^, whereas loss-of-function mutations protect against it^12^. Recent evidence demonstrates that PCSK9 also enhances the synthesis and secretion of triglyceride-rich lipoproteins^13^, another important risk factor for cardiovascular disease, which is associated with NAFLD development^14^. Accumulating data suggest that high intrahepatic or circulating PCSK9 levels play an important role in the storage and secretion of lipids (including triglycerides and fatty acids) in vivo^13,15,16^, contributing to the pathogenesis of NAFLD. More recently, our study showed that metformin treatment significantly decreased circulating PCSK9 and LDL-C levels^17^, which suggests the implication of PCSK9 signaling in metformin treatment for olanzapine-induced NAFLD. Herein, we studied the role of PCSK9 in olanzapine-induced NAFLD as well as in the therapeutic effect of metformin, elucidating the cellular and molecular mechanisms involved.

## Materials and methods

### Ethical statement

All animal experiments were performed according to the National Health Guidelines for Laboratory Care and Use and were approved by the Experimental Animal Ethics Committee of the Second Xiangya Hospital of Central South University. Animal experiments were designed and conducted in accordance with Animal Research: Reporting of In Vivo Experiments (ARRIVE) guidelines.

### Animal experiments

Eight-week-old female C57BL/6 J mice were purchased from Hunan Stryker Jingda Animal Co., Ltd. Mice were housed in a specific pathogen-free environment under controlled temperature (20–23 °C) and light conditions (12-h dark/light cycle), with a normal chow diet and water provided ad libitum. After one week of adaptation, the 24 individuals were randomly divided into four groups: (1) daily gavage of 10% DMSO as the control group (n = 6); (2) olanzapine-treated mice receiving olanzapine (LY170053, MCE, USA) daily at 6 mg/kg body weight (n = 6); (3) metformin-treated mice receiving metformin (PHR1084, Sigma, USA) daily at 100 mg/kg body weight; and (4) combination therapy with 6 mg/kg body weight olanzapine and 100 mg/kg body weight metformin daily. Olanzapine was prepared as described previously^18^, and metformin was dissolved in phosphate-buffered saline (PBS). The weight of mice was monitored weekly.

After 8 weeks of treatment based on groups, mice were fasted overnight, weighed, and euthanized via prompt dislocation of the neck vertebra under anesthesia. The liver was quickly dissected and rinsed with normal saline solution. Liver sections were fixed with 4% paraformaldehyde and embedded in paraffin for histological examination. The rest of the tissue was snap frozen in liquid nitrogen and stored at − 80 °C for analysis of gene/protein expression.

### Cell experiment

The immortalized human hepatoma cell line (HepG2 cells) and alpha mouse liver 12 cell line (AML12 cells) was purchased from the Cell Bank of the Institute of Biochemistry and Cell Biology (Shanghai, China). All cell lines were tested for mycoplasma contamination via a PCR-based assay. The cells were cultured in Dulbecco’s modified Eagle’s medium at 37 °C in an atmosphere containing 5% CO_2_. Four groups were included in the in vitro experiments: control group, 100 μM olanzapine group, 2 mM metformin group, and 100 μM olanzapine + 2 mM metformin group. Olanzapine was added to the medium at a final concentration of 100 μM based on the optimal concentration selected in previous experiments for 24 h^5^, and the final concentration of metformin was 2 mM. The DMSO treatment group served as the blank control group.

### Transient transfection

Plasmid constructs driving liver X receptor (LXR)α and PCSK9 overexpression were provided by Sino Biological, while PCSK9 siRNA and respective non-specific siRNAs were constructed by Shanghai Zorin Biological Technology Co. HepG2 cells used for transfection experiments were cultured to 70% confluence per dish in 6-well plates. Twenty-four hours after seeding the cells, the prepared transfection mixture was added to the well plate, mixed gently, incubated for 4–6 h, and the medium was then changed. Twenty-four hours after changing the medium, cell staining as well as protein and mRNA extraction were performed. The transfection procedure was repeated at least three times.

### Oil red O staining

The frozen liver sections of mice were stained with Oil Red O (ORO) using the Oil Red O staining solution (O0625, Sigma, USA) to assess hepatic steatosis. The tissues that had been cut into 6 μm slices were rinsed with 60% isopropyl alcohol. The samples were stained with a filtered ORO solution (0.5% isopropyl alcohol, then diluted with 60% distilled water) for 30 min. After washing twice, the slides were stained with hematoxylin. All images were captured using a microscope. The mean fat droplet size under ORO staining was calculated using Image J (version 1.8. 0). Intracellular lipid droplets of HepG2 cells and AML12 cells were also evaluated via ORO staining. HepG2 cells were rinsed twice with PBS, fixed with 4% formaldehyde solution for 30 min at 37 °C, and were then washed three times with PBS. The ORO dye solution was prepared according to the manufacturer's instructions. Staining was performed at 37 °C for 30 min. The slides were discolored in 60% isopropyl alcohol, and ORO staining was observed under a phase-contrast microscope. The absorbance was measured at 520 nm, and the results were analyzed using GraphPad Prism software.

### Hematoxylin and eosin (H&E) staining

Liver and adipose tissue samples were weighed. The samples were fixed in 10% neutral formalin overnight at 4 °C, dehydrated with ethanol, and embedded in paraffin. The paraffin sections were stained with H&E to observe the morphological characteristics of lipid accumulation.

### Western blotting analysis

HepG2 cells and AML12 cells were washed three times with ice-cold PBS. Standard RIPA lysis buffer (P0013B, Beyotime Biotechnology, China) containing 1% 0.5 mM PMSF was added to lyse the washed cells on ice for 30 min. After centrifugation at 13,000 rpm and 4 °C for 15 min, the supernatant was transferred to a new tube as a protein extract and quantified using a BCA protein concentration kit (CW0014S, CWBIO, China) according to the manufacturer’s instructions. The standard and diluted protein samples were added to 96-well plates, followed by the addition of BCA solution. The mixture was incubated at 37 °C for 30 min in the dark. Absorbance was measured using a microplate reader (DTX 880 Multimode Detector, Beckman Coulter, Brea, CA, USA) at 542 nm. The protein concentration of each sample was determined based on the plotted standard curve. Protein samples were diluted with loading buffer and PBS. The extracted proteins were separated via SDS-PAGE and transferred to a polyvinylidene difluoride (PVDF) membrane. The membranes were blocked with a rapid blocking solution (P0252, Beyotime, China). All blots were cut before hybridizing with the antibodies. Thereafter, the membranes were incubated in blocking buffer containing different primary antibodies against anti-LXRα (R24891, ZenBioScience, China), anti-PCSK9 (ab181142, Abcam, UK), and anti-tubulin (66,031–1-lg, Proteintech, USA) at 4 °C overnight. Subsequently, the membranes were washed three times with PBST for 10 min each to remove unbound antibodies. The washed membranes were then incubated with the corresponding HRP-conjugated secondary anti-mouse and anti-rabbit antibodies (SA00001-2, Proteintech, USA; SA00001-1, Proteintech, USA) diluted 1:5000 in milk/TBST for 1 h. As mentioned above, the membranes were washed with PBST three times, for 10 min each time. Finally, the protein expression levels were visualized with a Pierce™ ECL western blotting substrate (32,209, Thermo Scientific, USA) and quantified using a Molecular Imager ChemiDoc™ XRS + (Bio-Rad).

### Quantitative real-time PCR (qPCR) analysis

Total RNA was extracted from liver tissue samples or HepG2 cells / AML12 cells using an RNA extraction kit (K0731, Thermo Scientific, USA). Chloroform was then added to the samples, and the mixtures were layered into three layers: a colorless water phase layer (including RNA), an organic layer, and a red phenol layer. The colorless water phase layer was absorbed into a new EP tube, and an equal volume of 75% anhydrous ethanol was added. Total RNA was reverse-transcribed into cDNA using a Transcriptor First-Strand cDNA Synthesis Kit (K1622, Thermo, USA) according to the manufacturer’s instructions. SYBR Green Select Master Mix (172–5121, Bio-Rad, USA) was used for qPCR. GAPDH levels were used to normalize the mRNA expression levels of the target genes. The primers pairs for qPCR are listed in Tables 1 and 2.

**Table 1:** Oligonucleotide sequences of primers targeting mouse genes.

Gene	Primer	Sequence
Lxr	Forward	AAGTTCTCTGGACACTCCCG
	Reverse	TGGCTCTAAGATGACCACGA
Pcsk9	Forward	TTGCCCCATGTGGAGTACATT
	Reverse	GGGAGCGGTCTTCCTCTGT
Gapdh	Forward	ATGGGCGGAATGGTCTCTTTC
	Reverse	TGGGGACCTTGTCTTCATCAT
Acc	Forward	AGGTGGTGATAGCCGGTATGT
	Reverse	TGGGTAATCCATAGAGCCCAG
Fas	Forward	CACCTGCCTCTTCGGGATTT
	Reverse	TCTGAGAACTTGTGGTGGGC
Scd1	Forward	CTGATCCTGAGTAATGCAAGGTT
	Reverse	TGGATGCAATAATCACGCATGG
Dagt1	Forward	CATCATCGTGGTGGGAGGTG
	Reverse	TGGGAACCAGATCAGCTCCAT
Dagt2	Forward	AGGTGGTGATAGCCGGTATGT
	Reverse	TGGGTAATCCATAGAGCCCAG
Cpt1α	Forward	CTATGCGCTACTCGCTGAAGG
	Reverse	GGCTTTCGACCCGAGAAGA
Scad	Forward	CCGGCAGAACAAGGGTATCA
	Reverse	CCGGCAGTCCTCAAAGATGA
Pparα	Forward	TGGTGCATTTGGGCGTATCT
	Reverse	CACAGAGCGCTAAGCTGTGA
Acox1	Forward	TGAATCAGGGCACCACTGC
	Reverse	CTCGAAGATGAGTTCCGTGGC

**Table 2:** Oligonucleotide sequences of primers targeting human genes.

Gene	Primer	Sequence
LXR	Forward	CTTCTGGACAGGAAACTGCACC
	Reverse	TACCAAGGCACTGTCCAAATCC
PCSK9	Forward	CTCAGCTCCCGAGGTCATCA
	Reverse	CAGCCTGTGATGTCCCACTC
GAPDH	Forward	CCATGGGTGGAATCATATTGGA
	Reverse	TCAACGGATTTGGTCGTATTGG
ACC	Forward	GCTCGTGGATGAACCAGACT
	Reverse	CCACTTCCAAAAAGACCTAGCC
FAS	Forward	GTCTTGAACTCCTTGGCGGA
	Reverse	AGGAAGATAGCCATGCCGAG
SCD1	Forward	CCCGACGTGGCTTTTTCTTC
	Reverse	GCCAGGTTTGTAGTACCTCCT
DAGT1	Forward	CGGTCCCCAATCACCTCATC
	Reverse	AGACTCGGAGTTCCACCAGT
DAGT2	Forward	CCAGACAGCAACAAGACCGA
	Reverse	GAGCCCATGAAGGGTGTCAA
CPT1α	Forward	TCCAGTTGGCTTATCGTGGTG
	Reverse	TCCAGAGTCCGATTGATTTTTGC
SCAD(ACADS)	Forward	CGGCAGTTACACACCATCTAC
	Reverse	GCAATGGGAAACAACTCCTTCTC
PPARα	Forward	TTCGCAATCCATCGGCGAG
	Reverse	CCACAGGATAAGTCACCGAGG

The following genes were analyzed using qPCR: LXR, PCSK9, lipogenesis-associated genes (acetyl-CoA carboxylase [ACC], fatty acid synthase [FAS], stearoyl-coenzyme A desaturase 1 [SCD1], diacylglycerol acyltransferase 1 [DGAT1], and diacylglycerol acyltransferase 2 [DGAT2]), genes related to lipid β-oxidation (carnitine palmitoyl transferase 1α [CPT1α], acyl-CoA dehydrogenase short chain [SCAD], peroxisome proliferator activated receptor alpha [PPARα], and acyl-CoA oxidase 1 [ACOX1]).

### Immunofluorescence

Immunofluorescence was used to study the expression of PCSK9 in liver sections and cell lines. A rabbit PCSK9-specific antibody (ab185194, Abcam, England and bs-6060r, Bioss, China) was used as the primary antibody. Anti-rabbit IgG (Invitrogen) antibodies were used as the corresponding secondary antibodies. Immunofluorescence images were obtained using a fluorescence microscope (X173; Olympus, Japan).

### Cell activity detection

Cell Counting Kit 8 (CCK 8; K1018, APExBIO, USA) was used to detect cell viability. Briefly, 1000 cells per well were seeded in 96-well plates with 100 μL culture medium and treated with 0 or 100 μM olanzapine for 24 h. 10 μL CCK8 solution was added and incubated at 37 °C with 5% CO_2_ for 3 h. Absorbance was measured at 450 nm.

### Statistical analysis

Statistical analysis of all experimental data was performed using GraphPad Prism software (version 8.0) and SPSS (IBM version 17.0, SPSS Inc., USA). Data are shown as the mean ± SEM. One-way analysis of variance (ANOVA) was applied to compare the means of two groups followed by Dunnett’s post-hoc t-test for multiple comparisons. Statistical significance was set at *P* < 0.05.

## Results

### Metformin attenuates olanzapine-induced metabolic disturbance and hepatic/hepatocyte lipid accumulation in mice and cells

**Figure 1:**
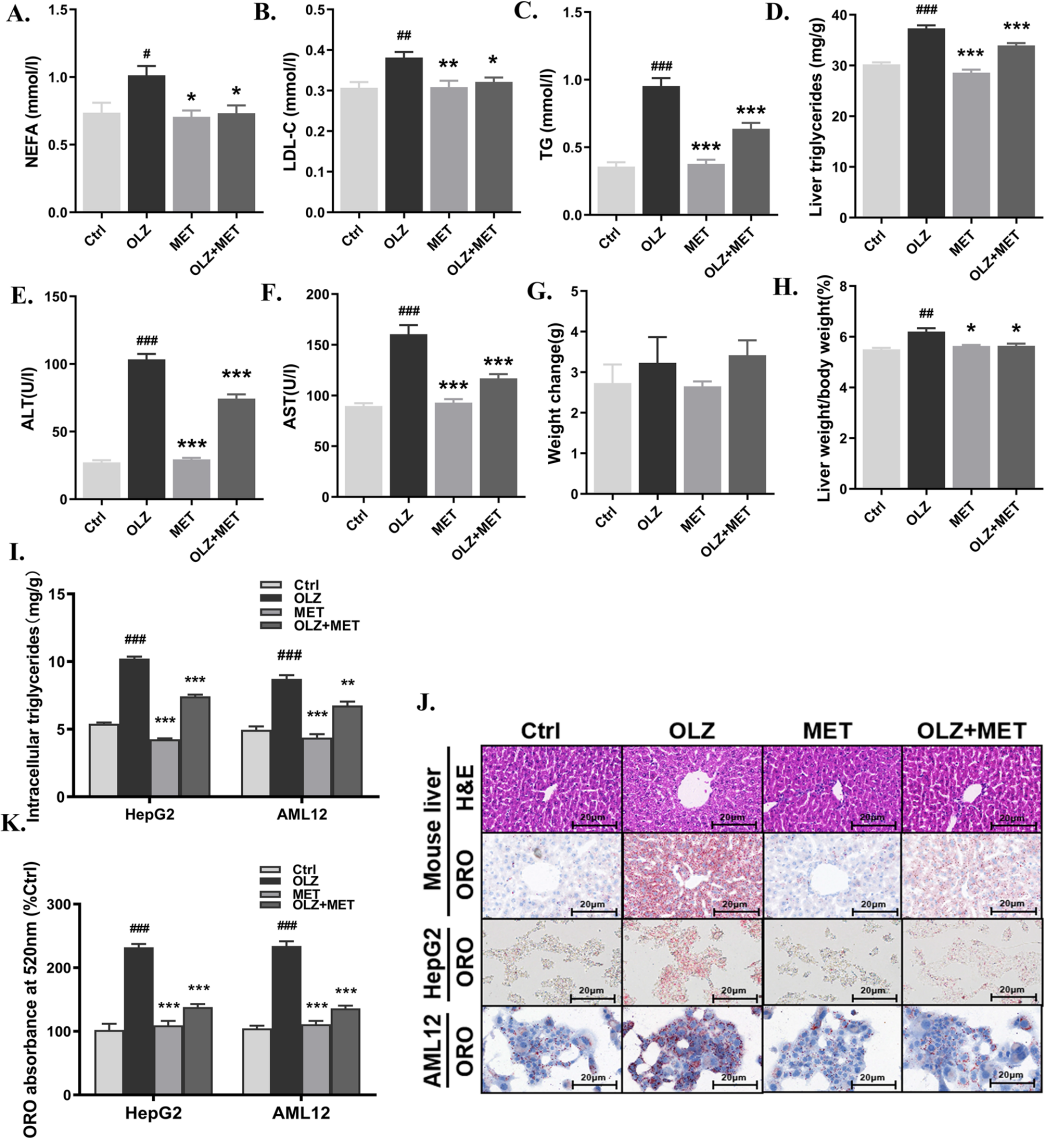
Metformin attenuates olanzapine-induced metabolic disturbance and hepatic/hepatocyte lipid accumulation in mice and cells. (**A**) Plasma NEFA levels. (**B**) Plasma LDL-C levels. (**C**) Plasma TG levels. (**D**) Liver triglyceride levels. (**E**) Plasma ALT levels. (**F**) Plasma AST levels. (**G**) Body weight. (**H**) Liver weight/body weight ratio. (**I**) Triglyceride levels in HepG2 cells and AML12 cells. (**J**) H&E and ORO staining. (**K**) ORO absorbance at 520 nm in HepG2 cells and AML12 cells. Ctrl, control; OLZ, olanzapine; MET, metformin. Data represent the mean ± SEM. ^#^*p* < 0.05, ^##^*p* < 0.01, ^###^*p* < 0.001 *vs.* control; *p < 0.05, **p < 0.01, ***p < 0.001 *vs.* olanzapine; one-way ANOVA plus Tukey’s post-hoc test.

To investigate the effect of metformin on the metabolic dysregulation induced by olanzapine, we administered olanzapine or metformin to mice and analyzed changes in metabolism and liver function. Olanzapine significantly upregulated plasma LDL-C, triglycerides and non-esterified fatty acid (NEFA) concentrations, while also increasing triglyceride content in the liver after 8 weeks of treatment (Fig. 1A–D). NEFA, LDL-C and triglyceride concentrations were significantly lower in the olanzapine + metformin co-treatment group (Fig. 1A–C). Moreover, olanzapine treatment increased plasma levels of alanine aminotransferase (ALT) and aspartate transaminase (AST) when compared to those in the control group (Fig. 1E,F). This effect was ameliorated via metformin co-treatment. In addition, although there was no significant difference in body weight between the groups, mice in the olanzapine group exhibited a significantly higher liver weight as well as liver/body weight ratio, both of which were improved via co-treatment with olanzapine and metformin (Fig. 1G,H). Experiments with HepG2 cells and AML12 cells confirmed that the olanzapine-induced increase in triglycerides was effectively eliminated via metformin co-treatment (Fig. 1I). And CCK8 assays further showed that 100 μM olanzapine caused a decrease of cell activity and proliferation in HepG2 and AML12 cells (Fig. 1 in Supplementary materials), which might be attributed to the cytotoxicity at such high doses of olanzapine treatment and the effect of olanzapine-induced hepatic steatosis.

Next, we performed H&E and ORO staining to observe the pathological alterations in mouse liver tissues. H&E staining indicated that, compared with the control group, hepatocytes in the olanzapine intervention group were deformed and compressed (Fig. 1J). Further, cytoplasmic lipid droplets varied in size and numbers. In addition, the amount and size of lipid droplets within hepatocytes were also greater. However, in the olanzapine + metformin group, most hepatocytes exhibited a normal ultrastructure, intact cell membrane, and less lipid droplet accumulation, as indicated via H&E staining (Fig. 1J). Metformin co-treatment significantly decreased positive ORO staining compared to olanzapine alone (Fig. 1J,K). In agreement with the in vivo results, metformin co-treatment reversed olanzapine-induced lipid accumulation in HepG2 cells and AML12 cells, significantly reducing the number of intracellular lipid droplets (Fig. 1I,J).

### Olanzapine-associated hepatic steatosis is associated with elevated PCSK9 levels

**Figure 2:**
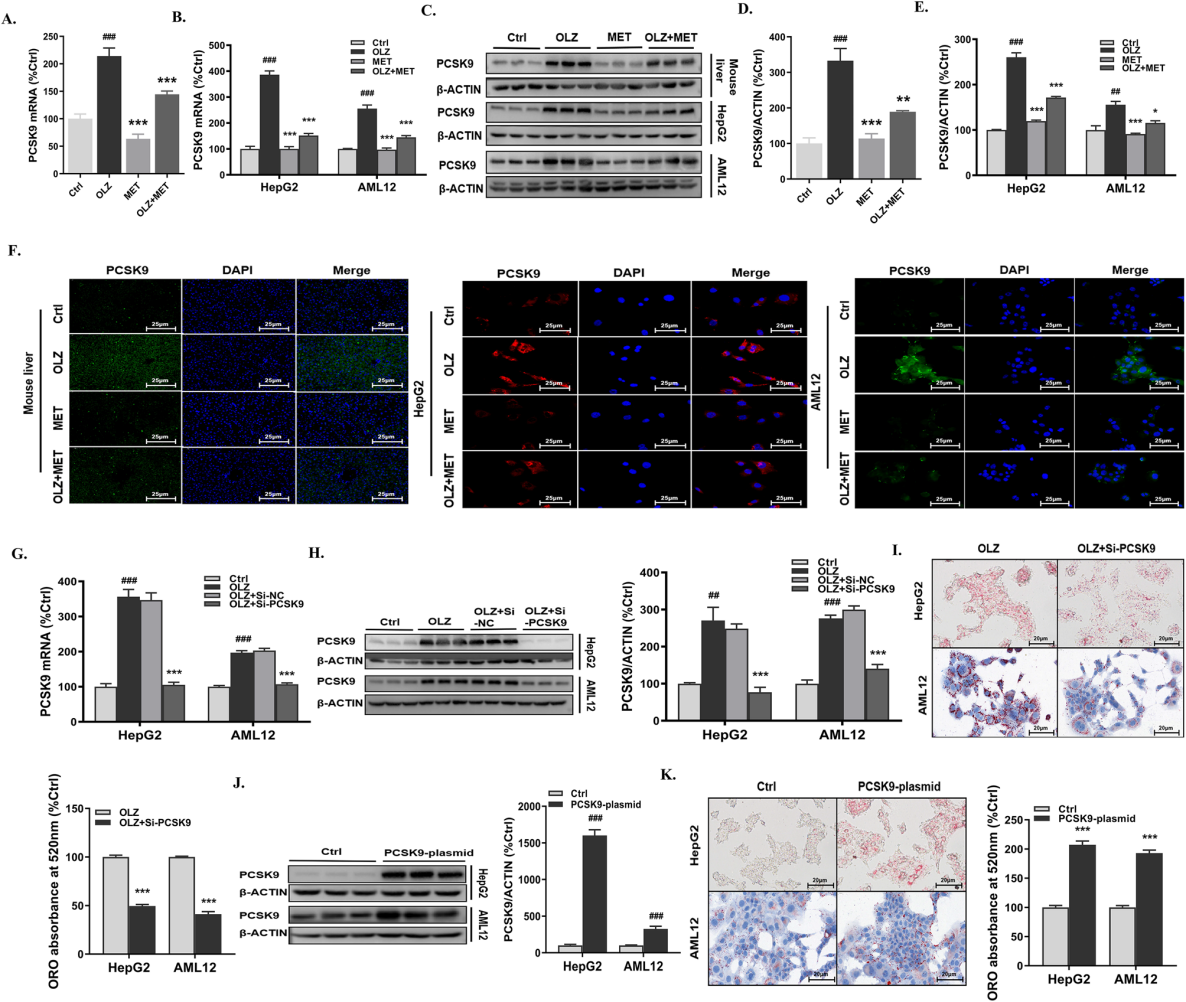
Olanzapine-associated hepatic steatosis is associated with elevated PCSK9 levels. PCSK9 mRNA in (**A**) mouse liver tissues, (**B**) HepG2 cells and AML12 cells. (**C-E**) PCSK9 protein levels in mouse liver tissues, HepG2 cells and AML12 cells. (**F**) Immunofluorescence staining of PCSK9 in mouse liver tissues, HepG2 cells and AML12 cells. PCSK9 (**G**) mRNA and (**H**) protein levels in HepG2 cells and AML 12 cells treated with si-PCSK9. (**I**) ORO staining of HepG2 cells and AML 12 cells treated with si-PCSK9. (**J**) PCSK9 protein levels in HepG2 cells and AML 12 cells treated with the PCSK9 plasmid. (**K**) ORO staining of HepG2 cells and AML 12 cells treated with the PCSK9 plasmid. Ctrl, control; OLZ, olanzapine; MET, metformin; Si-PCSK9, PCSK9 siRNA. Data represent the mean ± SEM. ^##^*p* < 0.01, ^###^*p* < 0.001 *vs.* control; **p < 0.01, ***p < 0.001 *vs.* olanzapine; Student’s two-tailed *t*-test and one-way ANOVA plus Tukey’s post-hoc test.

To investigate the role of PCSK9 in olanzapine-induced hepatic steatosis, PCSK9 mRNA and protein levels were measured. As shown in Fig. 2A,C,D, both the mRNA and protein expression of PCSK9 in liver tissues were significantly upregulated in the olanzapine group when compared to the control group. Further, immunofluorescence staining of hepatic PCSK9 revealed the same phenomenon (Fig. 2F). Consistently, olanzapine administration upregulated the mRNA and protein levels of PCSK9 in HepG2 cells and AML12 cells (Fig. 2B,C,E). The upregulation was also confirmed via immunofluorescence staining (Fig. 2F). To verify the exact role of PCSK9 in olanzapine-induced lipid accumulation, PCSK9 was knocked down or overexpressed in vitro (Fig. 2G–K). The pathological state of olanzapine-induced intracellular lipid accumulation was effectively improved in HepG2 cells and AML12 cells after successful *PCSK9* knockdown (Fig. 2G–I). Moreover, as indicated via ORO staining, overexpression of PCSK9 markedly increased the number and size of lipid droplets within hepatocytes (Fig. 2J,K). These results highlighted the essential role of PCSK9 in olanzapine-related lipid dysfunction.

### Metformin alleviates olanzapine-induced hepatic lipid accumulation by inhibiting PCSK9 and lipid synthesis-related gene expression in vivo and in vitro

**Figure 3:**
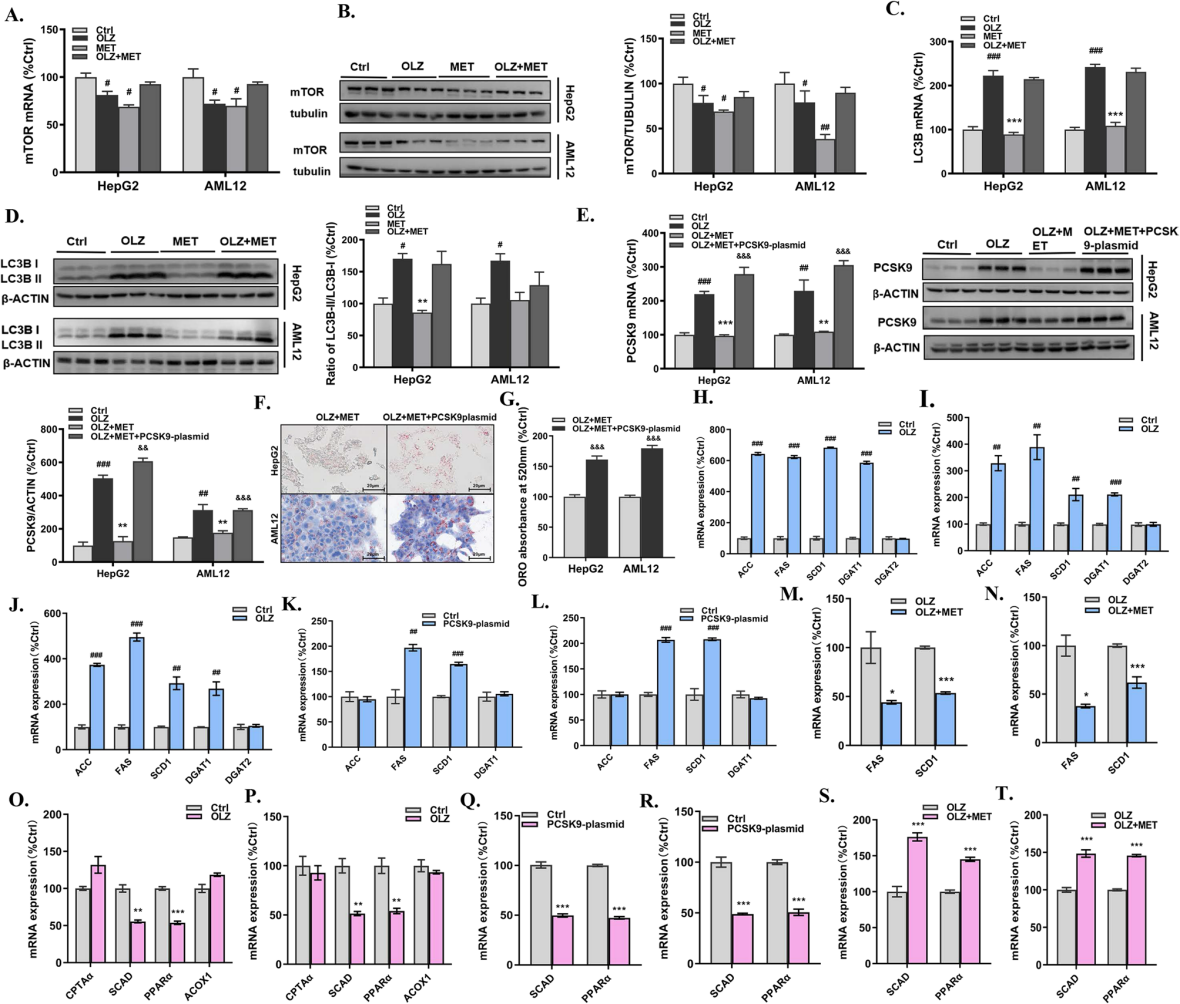
Metformin alleviates olanzapine-induced hepatic lipid accumulation by inhibiting PCSK9 and lipid synthesis-related gene expression in vivo and in vitro. mTOR (**A**) mRNA and (**B**) protein levels in HepG2 cells and AML12 cells. (**C**) LC3B mRNA levels in HepG2 cells and AML12 cells. (**D**) Ratio of LC3B II/LC3B I in HepG2 cells and AML12 cells. (**E**) PCSK9 mRNA and protein levels in HepG2 cells and AML12 cells treated with the PCSK9 plasmid. (**F, G**) ORO staining of HepG2 cells and AML12 cells treated with the PCSK9 plasmid. ACC, FAS, SCD1, DGAT1, and DGAT2 mRNA levels in (**H**) mouse liver tissues, (**I**) HepG2 cells and (**J**) AML12 cells. ACC, FAS, SCD1and DGAT1 mRNA levels in (**K**) HepG2 cells and (**L**) AML12 cells treated with the PCSK9 plasmid. FAS and SCD1mRNA levels in (**M**) HepG2 cells and (**N**) AML12 cells treated with the olanzapine and metformin. CPT1α, SCAD, PPARα, ACOX1 mRNA levels in (O) HepG2 cells and (**P**) AML12 cells treated with olanzapine; SCAD and PPARα mRNA levels in (Q) HepG2 cells and (R) AML12 cells treated with PCSK9 plasmid; SCAD and PPARα mRNA levels in (**S**) HepG2 cells and (**T**) AML12 cells treated with olanzapine and metformin. OLZ, olanzapine; MET, metformin. Data represent the mean ± SEM. ^##^*p* < 0.01, ^###^*p* < 0.001 *vs.* control; **p < 0.01, ***p < 0.001 *vs.* olanzapine; one-way ANOVA plus Tukey’s post-hoc test.

Accumulating evidence suggests that autophagy may represent a valuable target in NAFLD, because of its anti-steatogenic properties in hepatocytes via lipophagy, and its beneficial effects on the transition to NASH, via hepatoprotective effects of mitophagy in hepatocytes and anti-inflammatory properties in macrophages^19^. Since previous studies found metformin could enhance autophagy^20,21^, we conducted further experiments to explore the contribution of autophagy to the effects of metformin. Rapamycin (mTOR) signaling is the classical pathway to regulate autophagy^22^ and the modification process of LC3B is particularly critical for the formation of autophagosome [23.24]. From the results conducted in HepG2 and AML12 cells, olanzapine treatment caused an obvious decline in mTOR expression levels (Fig. 3A,B). In addition, enhanced LC3B in mRNA levels and the ratio of LC3B II/LC3B I was observed after olanzapine treatment (Fig. 3C,D). However, metformin co-treatment did not affect LC3B II/LC3B I and mTOR levels in comparison with olanzapine-only treatment.

Our in vivo and in vitro experiments confirmed that compared with the olanzapine group, metformin co-treatment significantly reduced the level of PCSK9 (Fig. 2C). ORO staining indicated that, in comparison to the olanzapine group, metformin co-treatment dramatically reduced olanzapine-induced hepatic lipid accumulation (Fig. 1J). Under PCSK9 overexpression, the effect of metformin on olanzapine-induced lipid accumulation in hepatocytes was offset (Fig. 3E–G). Taken together, these results suggested that intact PCSK9 expression was required for olanzapine-induced steatosis, and the beneficial effect of metformin on olanzapine-induced lipid accumulation was dependent on PCSK9.

Recent studies have suggested a positive correlation between serum PCSK9 levels and the expression of genes involved in de novo lipogenesis and β-oxidation^11^. Considering the fact that olanzapine markedly upregulated PCSK9 levels in hepatocytes, we sought to investigate whether PCSK9 is involved in the process of hepatic lipid accumulation through these genes. To this end, we evaluated the mRNA expression of ACC, FAS, SCD1, DGAT1, and DGAT2, which are involved in de novo lipogenesis. ACC, FAS, SCD1, and DGAT1 were significantly upregulated in olanzapine-treated mice as well as in HepG2 cells and AML12 cells (Fig. 3H–J). However, no significant differences were found in DGAT2 mRNA expression. In addition, when PCSK9 was overexpressed, only FAS and SCD1 were upregulated (Fig. 3K,L). These results indicated that among lipogenesis-related genes, only FAS and SCD1 were correlated with elevated PCSK9. Thus, the effects of PCSK9 in olanzapine-related hepatic steatosis may be mediated via the upregulation of FAS and SCD1.

In addition, some genes associated with β-oxidation (CPT1α, SCAD, PPARα, and ACOX1) are also included. We found that olanzapine treatment decreased SCAD and PPARα mRNA levels by ~ onefold (Fig. 3O,P). To explore factors directly targeted by PCSK9, we further measured their mRNA levels in HepG2 and AML12 cells transfected with PCSK9 plasmid. Notably, we observed decreases in SCAD and PPARα expression when PCSK9 overexpression (Fig. 3Q,R).

To confirm the metformin-induced downregulation of PCSK9 in olanzapine-associated hepatic steatosis models and explore the specific underlying mechanism for alleviating hepatic lipid accumulation, we determined the mRNA levels of lipid synthesis-related and β-oxidation-related genes in the olanzapine and metformin co-treatment group. The results indicated that, in comparison with the olanzapine group, an obvious decline in the mRNA levels of FAS and SCD1 as well as an increase in SCAD and PPARα were observed after metformin treatment (Fig. 3M,N,S,T), and the degree of decrease or increase was similar to that of change under *PCSK9* overexpression.

### Metformin improves olanzapine-induced hepatic/hepatocyte steatosis via downregulation of LXRα

**Figure 4:**
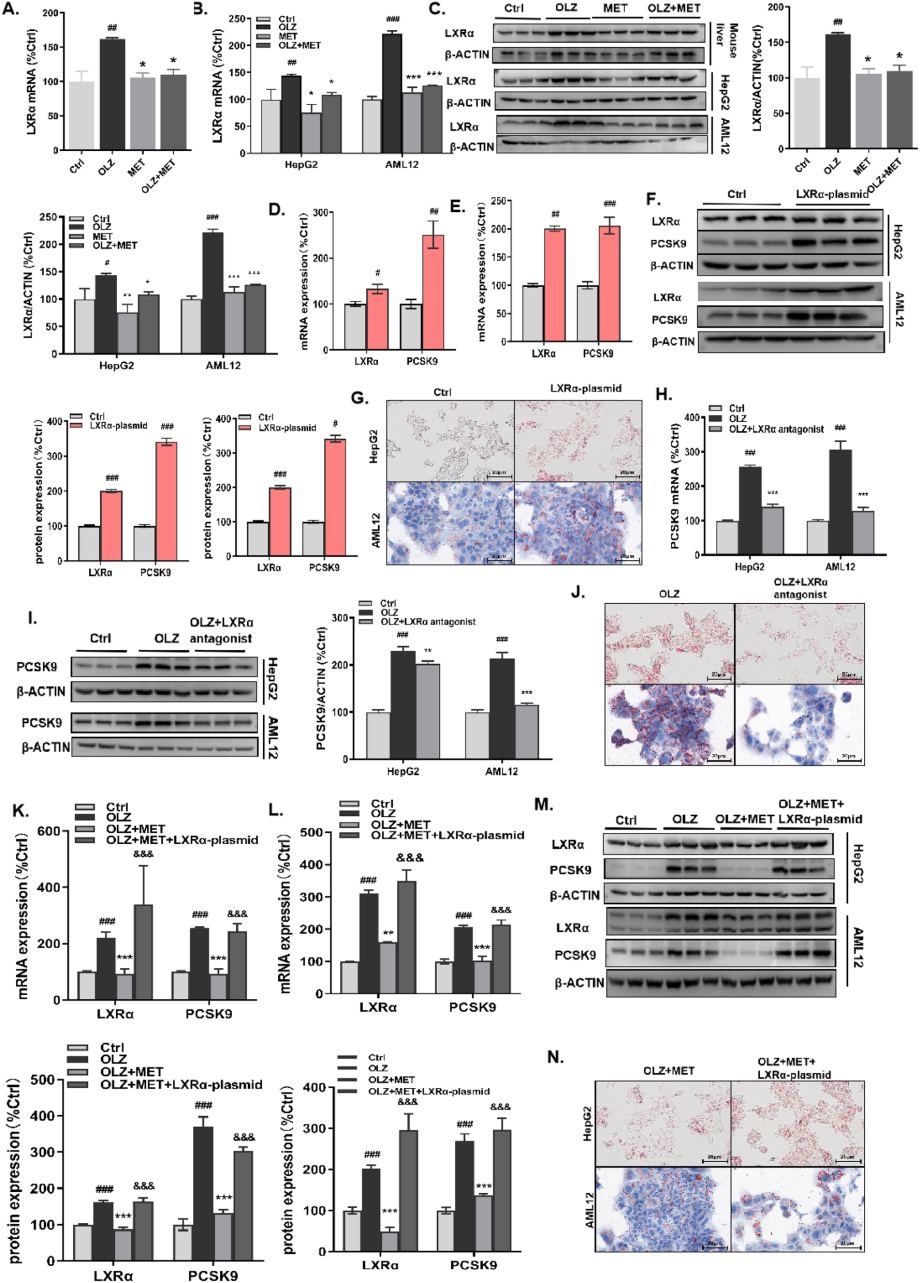
Metformin improves olanzapine-induced hepatic/hepatocyte steatosis via downregulation of LXRα. LXRα mRNA levels in (**A**) mouse liver tissues, (**B**) HepG2 cells and AML12 cells. LXRα protein levels in (**C**) mouse liver tissues, HepG2 cells and AML12 cells. LXRα and PCSK9 mRNA and protein levels in HepG2 cells (**D,F**) and AML12 cells (**E,F**) treated with the LXRα plasmid. (**G**) ORO staining of HepG2 cells and AML12 cells treated with LXRα plasmid. PCSK9 (**H**) mRNA and (**I**) protein levels in HepG2 cells and AML12 cells treated with an LXRα antagonist. (**J**) ORO staining of HepG2 cells and AML12 cells treated with an LXRα antagonist. PCSK9 mRNA and protein levels in (**K, M**) HepG2 cells and AML12 cells (**L, M**) treated with metformin and LXRα plasmid. (**N**) ORO staining of HepG2 cells treated with olanzapine, metformin and LXRα plasmid. Ctrl, control; OLZ, olanzapine; MET, metformin. Data represent the mean ± SEM. ^#^*p* < 0.05, ^##^*p* < 0.01, ^###^*p* < 0.001 *vs.* control; *p < 0.05, **p < 0.01, ***p < 0.001 *vs.* olanzapine; ^&^*p* < 0.05,^&&^*p* < 0.01, ^&&&^*p* < 0.001 *vs.* olanzapine + metformin; one-way ANOVA plus Tukey’s post-hoc test.

LXRs are nuclear receptors that regulate cholesterol and lipid homeostasis. Two subtypes of LXRs, LXRα and LXRβ, are associated with hepatic steatosis^25^. Accumulating evidence has suggested that both LXRα and β can promote lipogenesis by upregulating target genes, such as PCSK9 ^26^, sterol regulatory element-binding protein-1c (SREBP-1C)^27^, PPARγ^28^, carbohydrate response element-binding protein (ChREBP)^29^, SCD-1^30^, and lipoprotein lipase (LPL)^31^. Given the crucial role of LXRα in hepatic steatosis, its levels were determined in mouse livers as well as AML12 cells and HepG2 cells. Surprisingly, olanzapine treatment markedly upregulated LXRα at both the mRNA and protein levels (Fig. 4A–C). To further investigate whether LXRα plays a role in olanzapine-induced hepatic steatosis, LXRα antagonist treatment and LXRα overexpression were employed. ORO staining indicated that LXRα antagonist treatment significantly repressed olanzapine-induced lipid accumulation in HepG2 cells and AML12 cells (Fig. 4J), whereas LXRα overexpression exacerbated hepatic steatosis (Fig. 4G). Of note, we observed corresponding changes in PCSK9 levels. That is, LXRα antagonist treatment resulted in a marked downregulation of PCSK9 (Fig. 4H,I), while LXRα overexpression enhanced PCSK9 levels (Fig. 4D–F). Taken together, these results indicated that LXRα positively regulates PCSK9 during olanzapine-induced metabolic dysfunction.

To confirm the effect of olanzapine and metformin co-treatment on liver lipid metabolism via the LXRα pathway as well as to verify whether the level of PCSK9 was affected after treatment, we detected PCSK9 in HepG2 cells as well as AML12 cells and performed ORO staining. Compared with the olanzapine group, the protein and mRNA levels of LXRα and PCSK9 in the olanzapine + metformin group were strikingly decreased as was intracellular lipid accumulation (Fig. 4K–N). Additionally, when LXRα was overexpressed, PCSK9 was upregulated at both the mRNA and protein level (Fig. 4M). Meanwhile, the amount and size of the lipid droplets in HepG2 cells and AML12 cells increased slightly (Fig. 4N).

## Discussion

To the best of our knowledge, this study is the first to establish the role of PCSK9 in olanzapine-induced NAFLD. That is, olanzapine upregulates PCSK9 via LXRα signaling, thereby promoting the expression of target hepatic lipogenesis-associated genes, which contributes to lipid accumulation in the liver and, consequently, the development of NAFLD. Further, our data confirmed that the therapeutic effect of metformin on olanzapine-induced NAFLD was mediated via inhibition of the LXRα/PCSK9 axis. In particular, metformin inhibited PCSK9 expression by repressing LXRα, resulting in the downregulation of hepatic lipogenesis-associated genes, which in turn ameliorated olanzapine-induced NAFLD.

Olanzapine is the mainstay drug for schizophrenia owing to its superior efficacy and minor side effects^32^. Nevertheless, patients with schizophrenia receiving long-term olanzapine treatment are under great risk of developing metabolic disorders, such as weight gain, glucose abnormalities, and lipid metabolism dysregulation^33,34^. These side effects have complicated schizophrenia treatment, leading to higher rates of adverse cardiovascular events. Rodents receiving olanzapine are also predisposed to NAFLD, as confirmed by other studies and our previous data^4,5^. In the current work, olanzapine treatment led to hepatic steatosis in vivo and in vitro, characterized by excessive hepatic lipid accumulation and increased intra-hepatocyte lipid droplets. Thus, NAFLD is a major challenge in olanzapine treatment, increasing the morbidity and mortality associated with liver and cardiovascular diseases in schizophrenia patients.

Olanzapine-induced NAFLD is a consequence of the metabolic dysregulation caused by the drug. Dyslipidemia plays a key role in NAFLD pathogenesis, which is characterized by hepatic lipid dysregulation, including increased lipogenesis and decreased lipolysis within the liver^35^. In turn, these lead to the accumulation of intrahepatic neutral lipids. Approximately 40% of administered olanzapine is metabolized in the liver, yielding highly reactive oxidative metabolites^36^, which may damage hepatocytes. In this study, we chose PCSK9, the most documented regulator of lipid metabolism in recent years^37–39^, as the key factor to be investigated in relation to the underlying mechanism of olanzapine-induced NAFLD.

Furthermore, our study is the first to support the key role of PCSK9 signaling in olanzapine-induced NAFLD. PCSK9 is a well-established regulator of cholesterol homeostasis^37^. PCSK9 is mainly expressed and secreted by hepatocytes. When expressed on the surface of liver cells, PCSK9 binds to LDLR and is internalized as a PCSK9-LDLR complex, with LDLR transported to intra-hepatocyte lysosomes for degradation^40^. LDLR, a cell surface receptor predominantly expressed in the liver, is responsible for the endocytosis of LDL (the major transporter of circulating cholesterol) and its subsequent delivery to lysosomes for degradation, which results in reduced circulating LDL-C levels^41^. Thus, the PCSK9-mediated degradation of LDLR increases LDL-C levels and promotes cardiovascular disease^42^. Interestingly, recent data have identified PCSK9 as a trigger for NAFLD^11,43^. Of note, the recent study shows that olanzapine treatment induces PCSK9-mediated dyslipidemia in patients with schizophrenia^44^. Our current findings indicated that olanzapine upregulated the expression of hepatic PCSK9 accompanied by hepatic/hepatocyte steatosis in vivo and in vitro, which is characterized by the increased size and number of lipid droplets within hepatocytes. Conversely, knocking out the PCSK9 gene in hepatocytes in vitro effectively ameliorated hepatocyte steatosis, as indicated by reduced fat accumulation and lipid droplets in HepG2 cells and AML12 cells. Altogether, our findings establish the pathophysiological role of PCSK9 in olanzapine-induced NAFLD.

The efficacy of metformin in olanzapine-induced NAFLD was also confirmed in the current study. Metformin has previously been reported to attenuate olanzapine-induced metabolic disorders, including weight gain, type 2 diabetes, insulin resistance, and dyslipidemia^45,46^, all of which promote the development of NAFLD. Our previous findings^9^ and those of others^10^ have demonstrated the therapeutic effect of metformin on olanzapine-induced NAFLD. Consistently, the current study confirmed that metformin effectively reversed olanzapine-induced hepatocyte steatosis, as indicated by the alleviation of fat accumulation and the reduction of lipid droplets in vivo and in vitro.

Of note, our data identified PCSK9 as a novel target of metformin in olanzapine-induced NAFLD. As previously mentioned, PCSK9 is involved in the upregulation of triglyceride-rich lipoproteins and triglycerides through both LDLR-dependent and -independent pathways^13^. In addition, it has been demonstrated that high intrahepatic and circulating PCSK9 levels promote hepatic lipogenesis and lipid storage^11,43^, thus accelerating NAFLD pathogenesis. In this study, we demonstrated that metformin inhibits hepatic PCSK9 expression, in parallel to improving olanzapine-induced hepatic/hepatocyte steatosis in vivo and in vitro. Conversely, overexpression of PCSK9 in vitro dramatically attenuated the effects of metformin on hepatocyte steatosis. To our knowledge, this is the first report on the involvement of PCSK9 in olanzapine-induced NAFLD as well as in the therapeutic effect of metformin for this condition. To be noticed, metformin induces mitochondrial autophagy and endoplasmic reticulum autophagy through different pathways^20,21^, and damaged autophagy may represent a valuable target in NAFLD. However, from the results of increased ratio of LC3B II/LC3B I and decreased mTOR levels in this study, of which the former proves positive regulate factor of autophagy and the latter is negative^22–24^, autophagy seems not contribute to olanzapine-induced hepatic steatosis. Moreover, there was no difference in ratio of LC3B II/LC3B I and mTOR levels between metformin co-treatment with olanzapine and olanzapine mono-treatment group, which may exclude the action of autophagy on therapeutical effect of metformin. Thus, PCSK9 represents a potential therapeutic target for lipid and glucose metabolism disorders^43^ caused by atypical antipsychotics.

To screen the PCSK9 downstream factors involved in the effects of metformin on olanzapine-induced NAFLD, we determined the expression of a series of hepatic lipid metabolism-associated genes, including ACC, FAS, SCD1, DGAT1, DGAT2, CPT1α, SCAD, PPARα, and ACOX1. Olanzapine treatment induced a significant upregulation of ACC, FAS, SCD1, and DGAT1 as well as downregulation of SCAD and PPARα in vivo and in vitro. However, only FAS and SCD1 were considerably upregulated under PCSK9 overexpression, suggesting that these are downstream factors of PCSK9 in NAFLD. And SCAD and PPARα decreased when PCSK9 overexpression. Conversely, metformin administration attenuated the olanzapine-induced upregulation of PCSK9, FAS, and SCD1 as well as downregulation of SCAD and PPARα, effectively improving hepatic/hepatocyte steatosis. Thus, these data suggest that FAS, SCD1, SCAD and PPARα, as factors downstream of PCSK9, mediated the effects of metformin in olanzapine-induced NAFLD. That is, metformin ameliorated olanzapine-induced NAFLD by inhibiting the PCSK9-FAS/SCD1 or SCAD/PPARα axis.

Finally, we investigated the signaling pathways underlying the effects of metformin on olanzapine-induced NAFLD. *Hu *et al*.* found that metformin can reduce the serum PCSK9 levels which is consistent with our findings^17^. Considering that AMP-activated protein kinase (AMPK) signaling is the classic cascade targeted by metformin, they explored whether activation of the AMPK pathway contributes to the downregulation of metformin on PCSK9 levels. However, different from the action of metformin on PCSK9, activation of AMPK pathway by AMPK agonists did not affect PCSK9 expression in mRNA and protein levels as well as the secreted PCSK9^17^, suggesting that metformin-mediated downregulation of PCSK9 could not be attributable to AMPK activation. Besides, this study confirmed that metformin can reduce PCSK9 levels through suppressing hepatic intracellular glucose sensor, carbohydrate-responsive element-binding protein (ChREBP). Therefore, we believe that the effects of metformin on PCSK9 are AMPK-independent. Another previous study also indicated that olanzapine had no effect on AMPK activation in rats^4^. Thus, AMPK was not considered in the present study. Instead, we investigated LXRα, a key lipid-sensing nuclear receptor predominantly expressed in the liver, as a potential mediator of the effects of metformin. LXRα is an activator of the promoter of SREBP-1c^47^, and SREBP-1c has been well-documented as a key stimulator of hepatic lipogenesis and lipid accumulation, thereby promoting the development of NAFLD^48–50^. Of note, our studies^17^ and those of others^51–53^ have demonstrated that SREBP-1c upregulates PCSK9 expression and induces lipogenesis-associated genes, which are believed to accelerate NAFLD pathogenesis. In this study, olanzapine induced an upregulation of hepatic LXRα and PCSK9 expression, accompanied by fat accumulation and steatosis in vivo and in vitro. In contrast, LXRα antagonist treatment profoundly suppressed PCSK9 expression, effectively reversing olanzapine-induced fat accumulation and steatosis in vitro. However, overexpression of LXRα dramatically upregulated hepatocyte PCSK9 expression, stimulating intracellular fat accumulation in vitro, which indicated that LXRα was the regulate factor of PCSK9 consistent with previous studies^54,55^. Of note, metformin ameliorated the olanzapine-induced upregulation of LXRα and PCSK9, thus contributing to the reduction of hepatic fat accumulation and steatosis in vivo and in vitro. However, LXRα overexpression in hepatocytes robustly attenuated the beneficial effect of metformin. Taken together, our data highlight LXRα as a key factor in the treatment of olanzapine-induced NAFLD.

**Figure 5:**
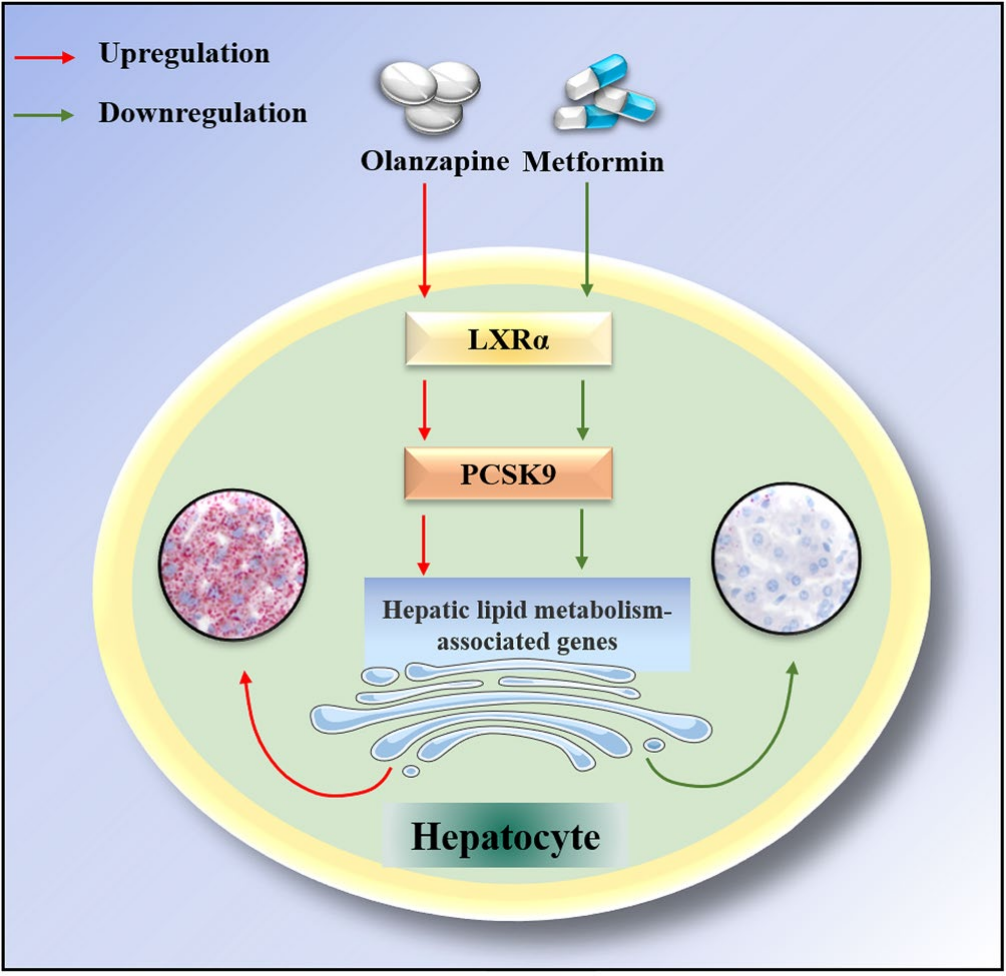
Mechanism underlying the therapeutic effects of metformin on olanzapine-induced NAFLD. Olanzapine induces the expression of LXRα in the liver and subsequently upregulates PCSK9 expression, which in turn affects hepatic lipid metabolism-associated genes (such as FAS, SCD1, SCAD and PPARα), thereby accelerating lipid accumulation. Conversely, metformin reduces the expression of LXRα in the liver and subsequently downregulates PCSK9, which in turn reduces the expression of FAS and SCD1, thereby ameliorating olanzapine-induced NAFLD.

Herein, we propose a novel mechanism underlying the therapeutic effects of metformin on olanzapine-induced NAFLD (Fig. 5). Olanzapine induces the expression of LXRα in the liver and subsequently upregulates PCSK9 expression, which in turn affects the expression of hepatic lipid metabolism-associated genes (such as FAS, SCD1, SCAD and PPARα), thereby accelerating lipid accumulation. Thus, olanzapine administration promotes NAFLD through the LXRα/PCSK9 axis. Conversely, metformin ameliorates olanzapine-induced NAFLD by inhibiting the LXRα/PCSK9 axis. To be noticed, this study was only done in female mice and that, as extensively described in the literature, the effects of antipsychotic agents might be different in male animals.

## Conclusion

The current study is the first to establish the role of PCSK9 in the protective effects of metformin against olanzapine-induced NAFLD. Our findings indicate that olanzapine promotes hepatic PCSK9 expression by upregulating LXRα, thereby affecting the expression of hepatic lipogenesis-associated genes FAS and SCD1 as well as oxidation-related genes SCAD and PPARα, which results in lipid accumulation and the development of NAFLD. In contrast, metformin effectively ameliorated olanzapine-induced NAFLD through the LXRα/PCSK9 axis. Our findings suggest that PCSK9 might serve as a therapeutic target in schizophrenia patients with olanzapine-induced NAFLD.

## Supplementary Information

Supplementary Information.
