# Supplementary material for: Metformin Ameliorates Hepatic Steatosis induced by olanzapine through inhibiting LXRα/PCSK9 pathway
Source: Sci Rep. 2022 Apr 4;12:5639. doi: 10.1038/s41598-022-09610-1 (PMC8979948; doi:10.1038/s41598-022-09610-1)
Supplement: Supplementary file 1 — Supplementary Information. [file 41598_2022_9610_MOESM1_ESM.pptx]

## Slide 1
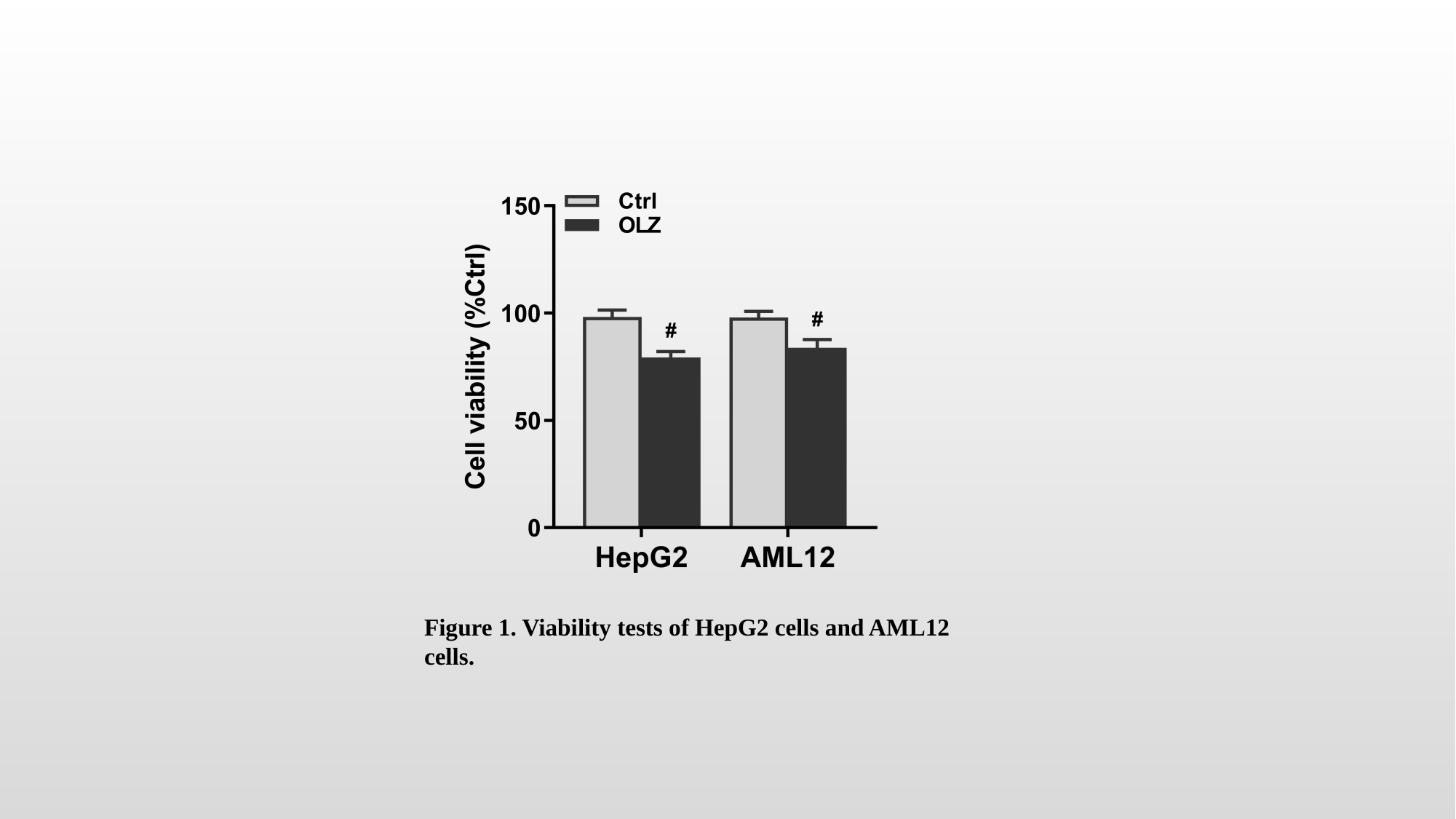

Figure 1. Viability tests of HepG2 cells and AML12 cells.

## Slide 2
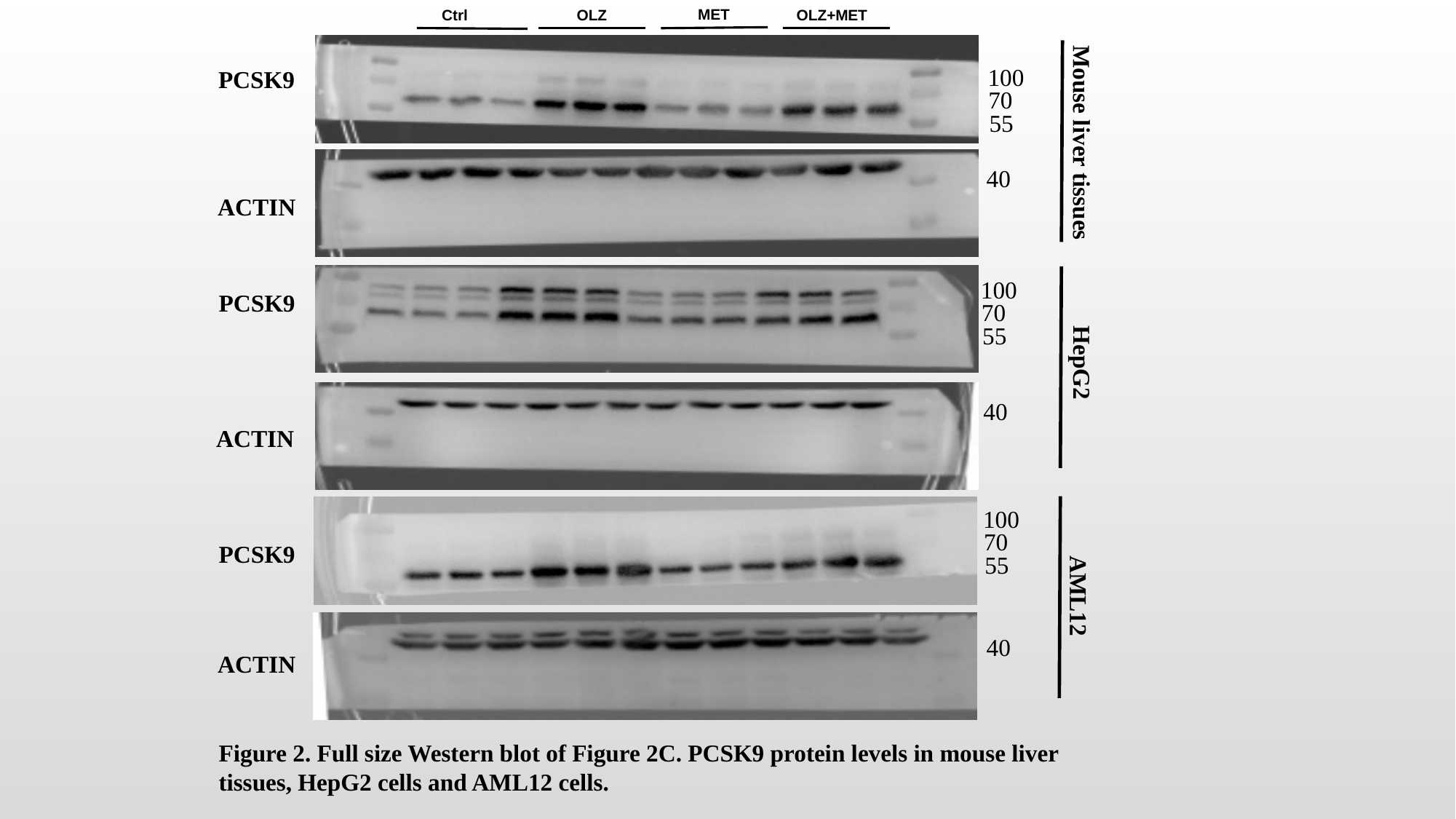

MET
Ctrl
OLZ
OLZ+MET
100
PCSK9
70
55
40
ACTIN
Mouse liver tissues
100
PCSK9
70
55
HepG2
40
ACTIN
100
70
PCSK9
55
AML12
40
ACTIN
Figure 2. Full size Western blot of Figure 2C. PCSK9 protein levels in mouse liver tissues, HepG2 cells and AML12 cells.

## Slide 3
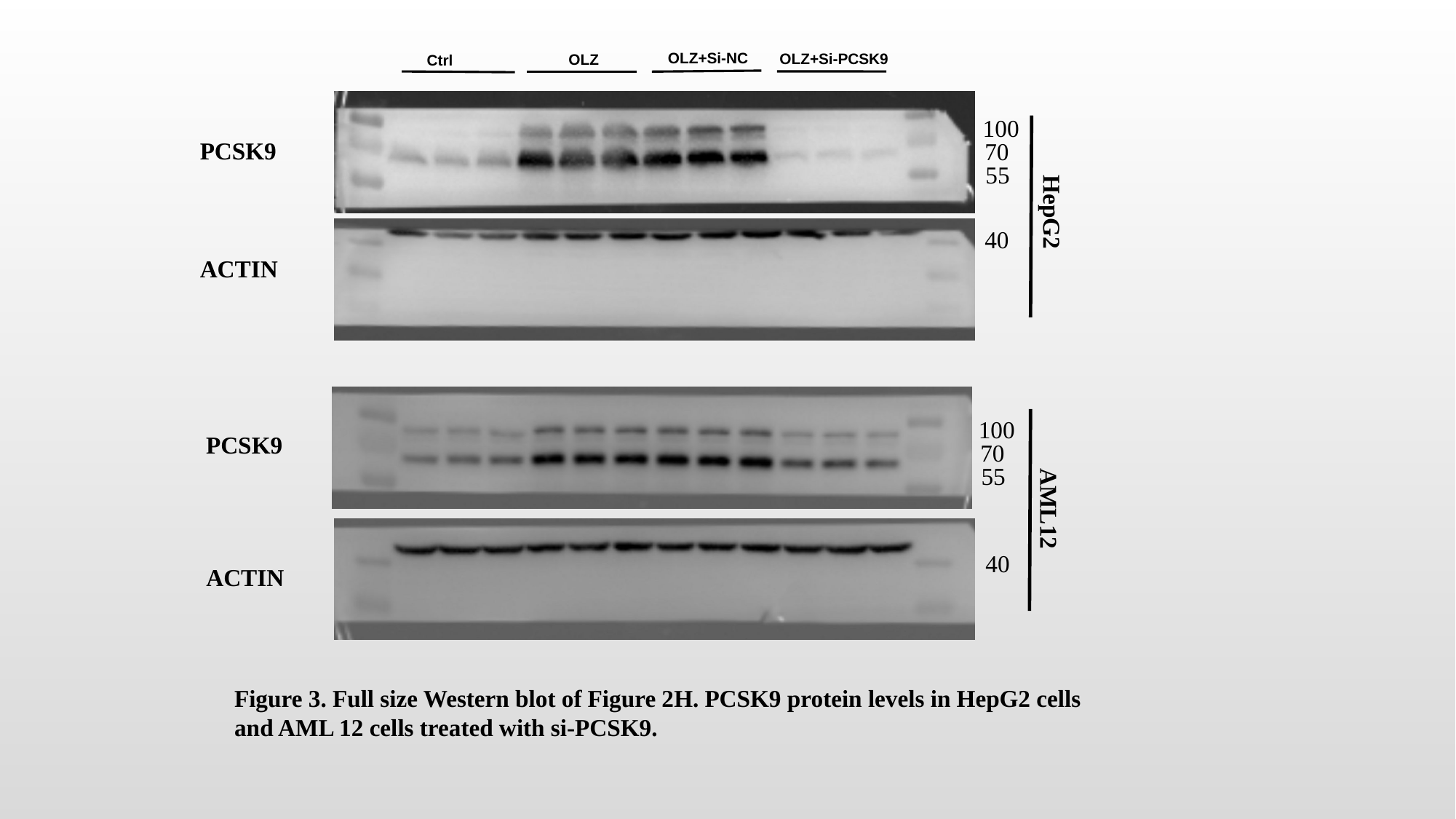

OLZ+Si-NC
OLZ+Si-PCSK9
OLZ
Ctrl
100
PCSK9
70
55
HepG2
40
ACTIN
100
PCSK9
70
55
AML12
40
ACTIN
Figure 3. Full size Western blot of Figure 2H. PCSK9 protein levels in HepG2 cells and AML 12 cells treated with si-PCSK9.

## Slide 4
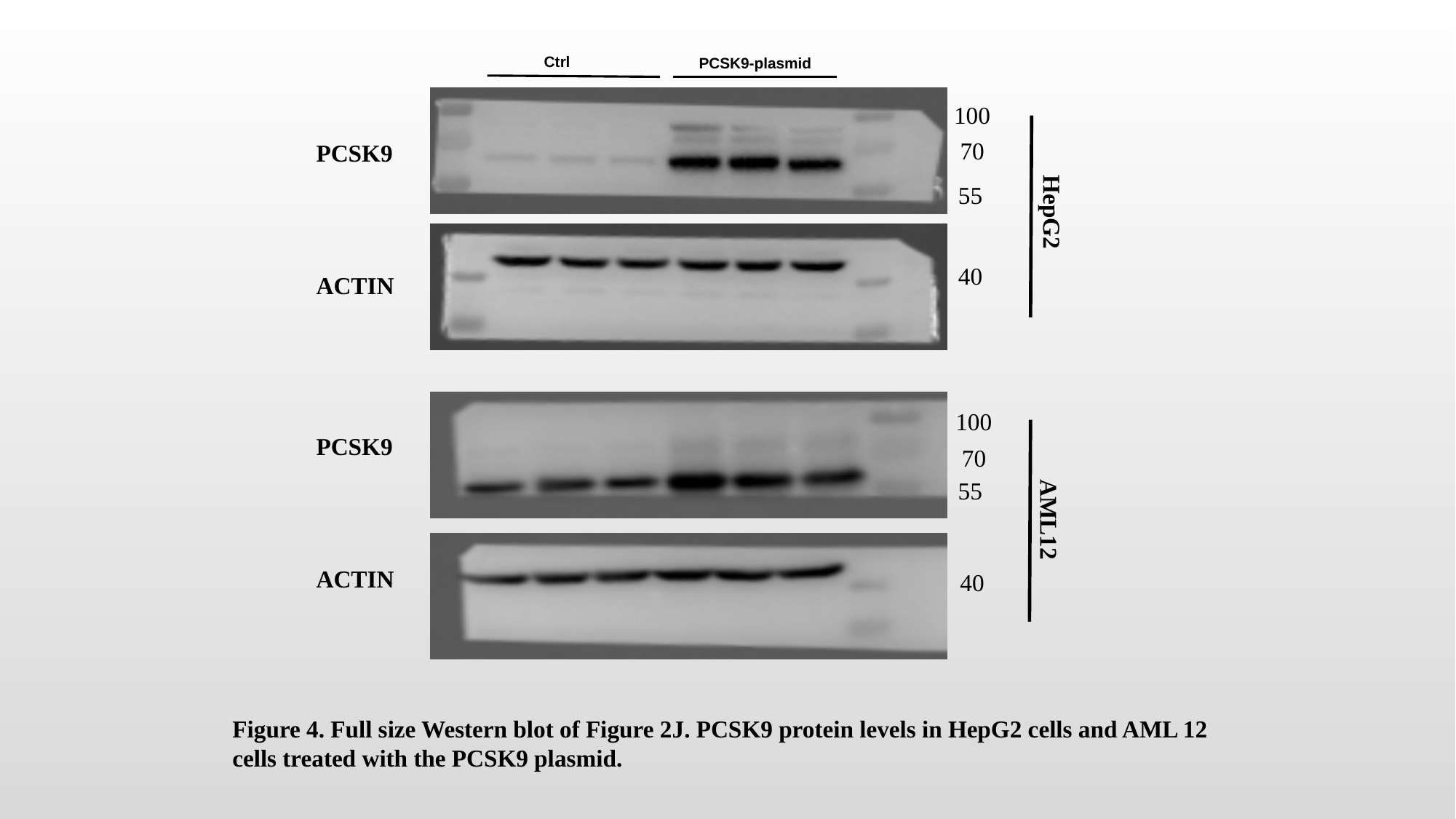

Ctrl
PCSK9-plasmid
100
70
PCSK9
55
HepG2
40
ACTIN
100
PCSK9
70
55
AML12
ACTIN
40
Figure 4. Full size Western blot of Figure 2J. PCSK9 protein levels in HepG2 cells and AML 12 cells treated with the PCSK9 plasmid.

## Slide 5
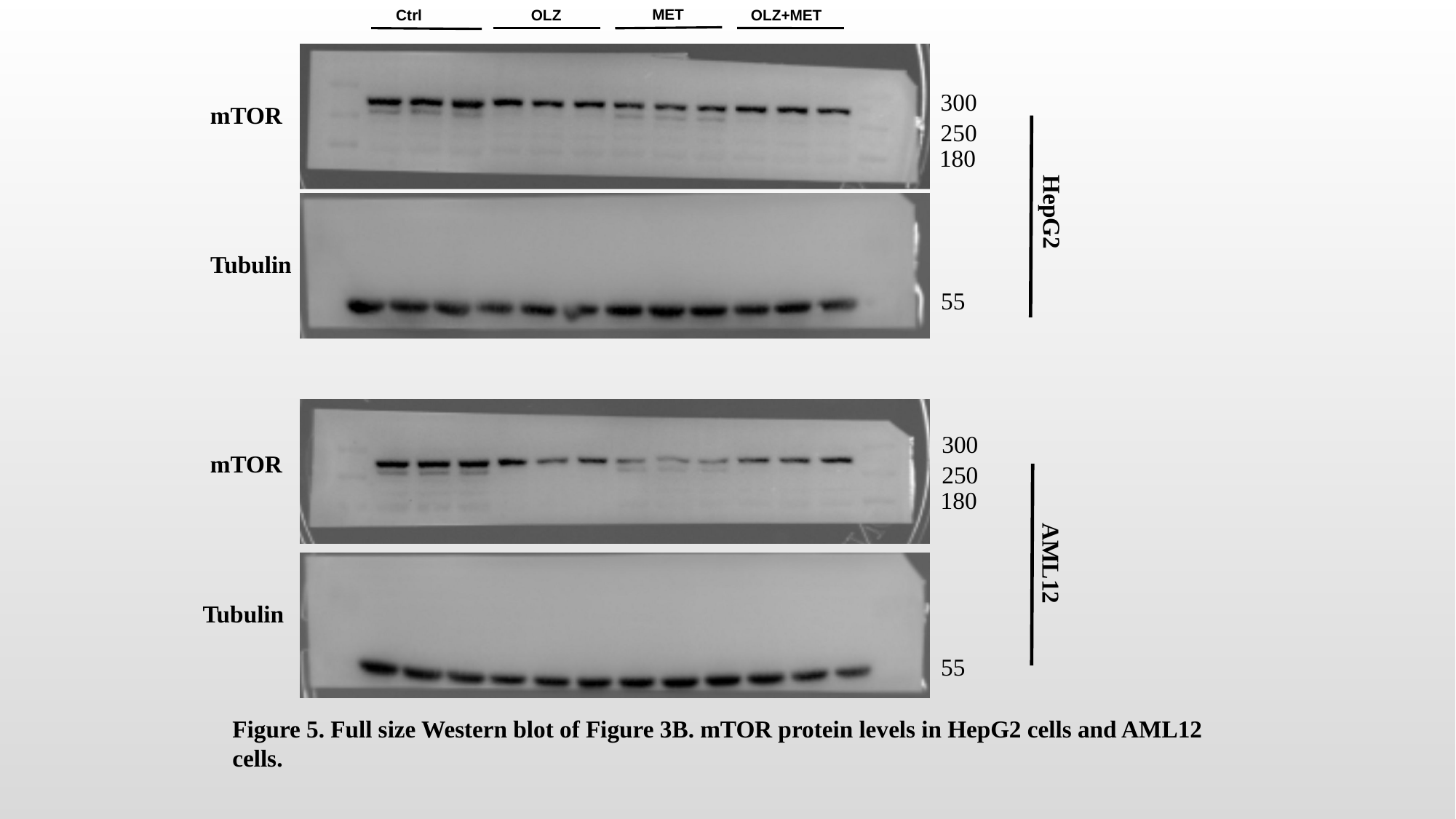

MET
Ctrl
OLZ
OLZ+MET
300
mTOR
250
180
HepG2
Tubulin
55
300
mTOR
250
180
AML12
Tubulin
55
Figure 5. Full size Western blot of Figure 3B. mTOR protein levels in HepG2 cells and AML12 cells.

## Slide 6
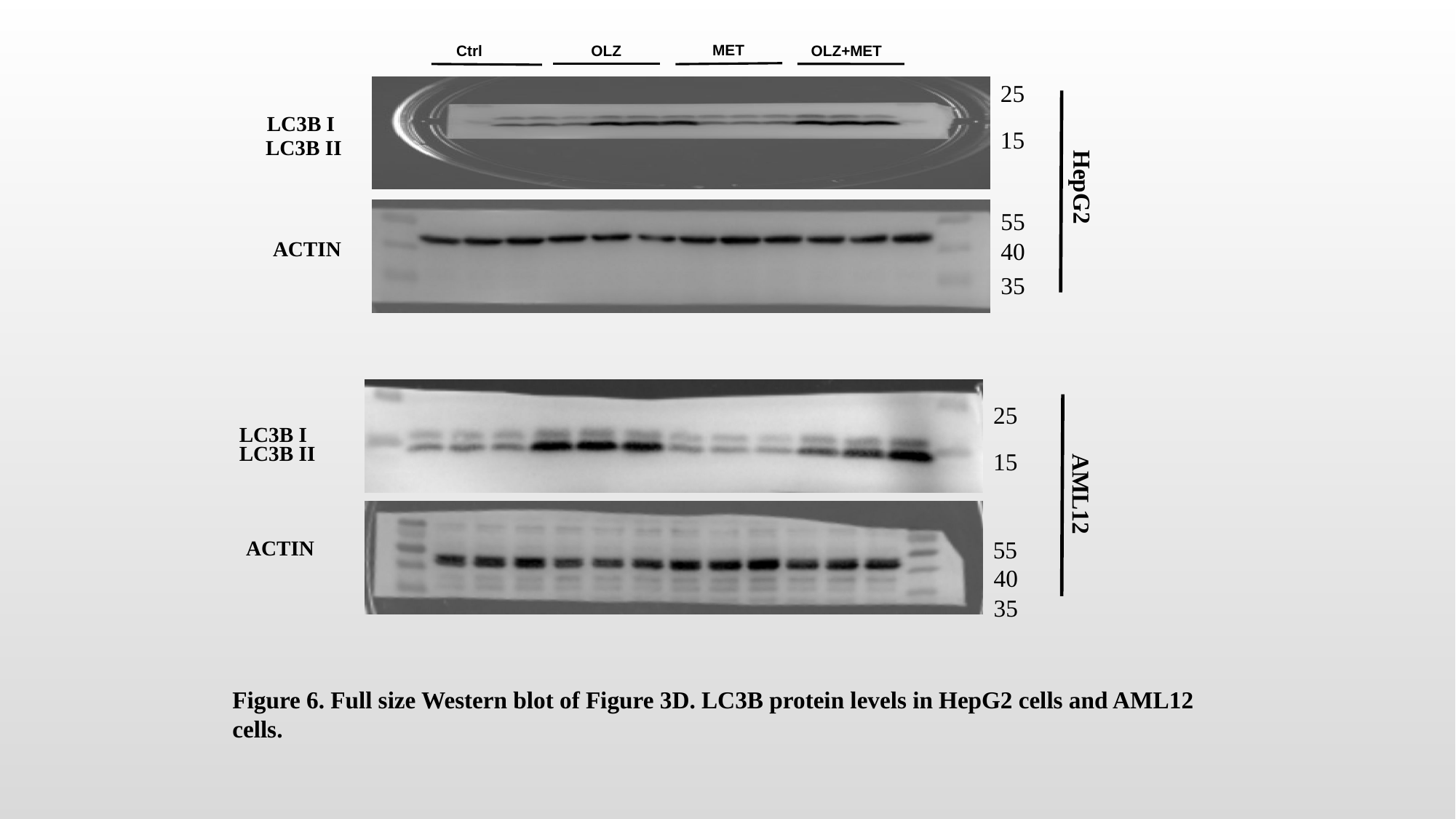

MET
Ctrl
OLZ
OLZ+MET
25
LC3B I
15
LC3B II
HepG2
55
ACTIN
40
35
25
LC3B I
LC3B II
15
AML12
55
ACTIN
40
35
Figure 6. Full size Western blot of Figure 3D. LC3B protein levels in HepG2 cells and AML12 cells.

## Slide 7
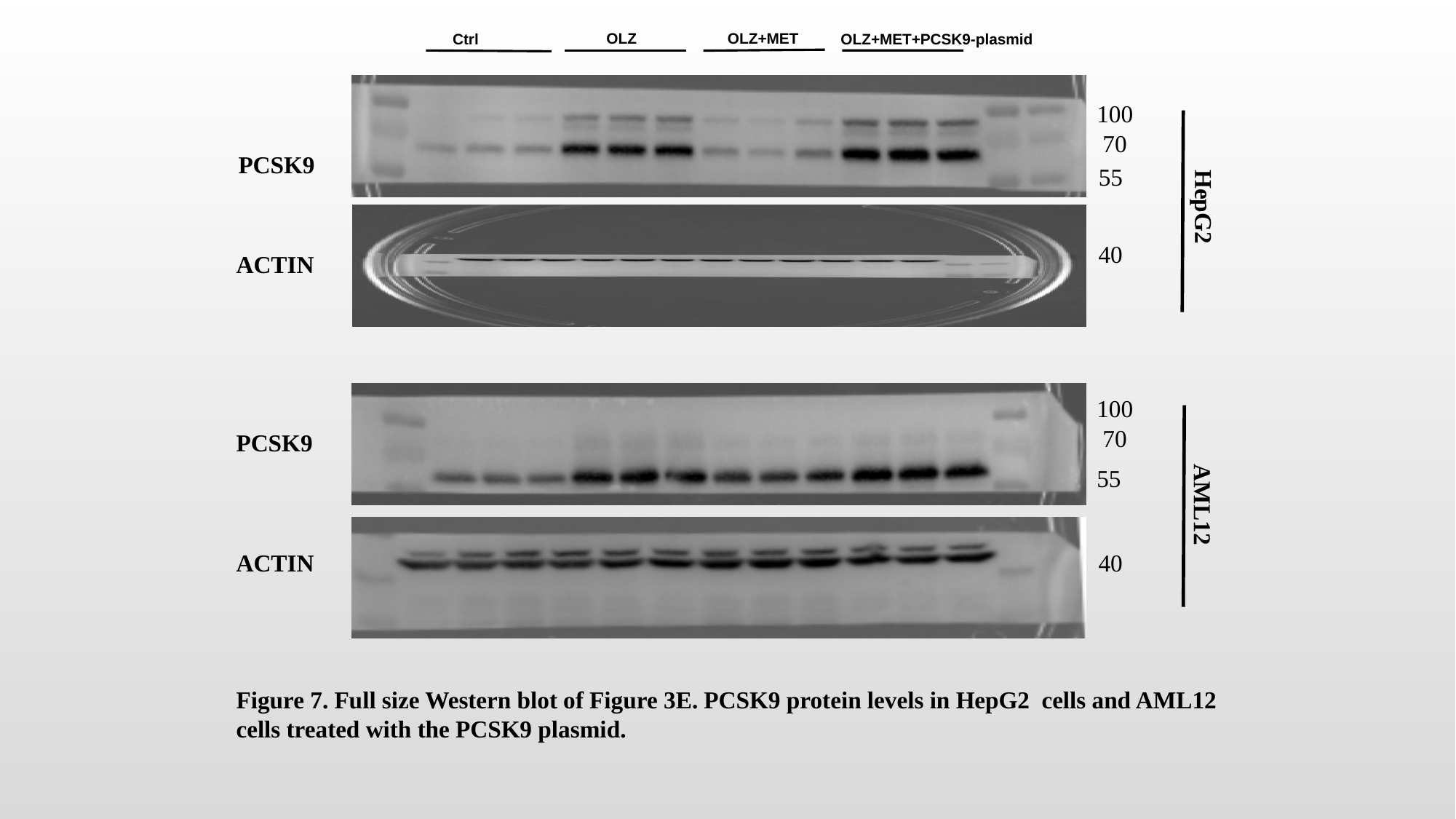

OLZ+MET
OLZ
OLZ+MET+PCSK9-plasmid
Ctrl
100
70
PCSK9
55
HepG2
40
ACTIN
100
70
PCSK9
55
AML12
ACTIN
40
Figure 7. Full size Western blot of Figure 3E. PCSK9 protein levels in HepG2 cells and AML12 cells treated with the PCSK9 plasmid.

## Slide 8
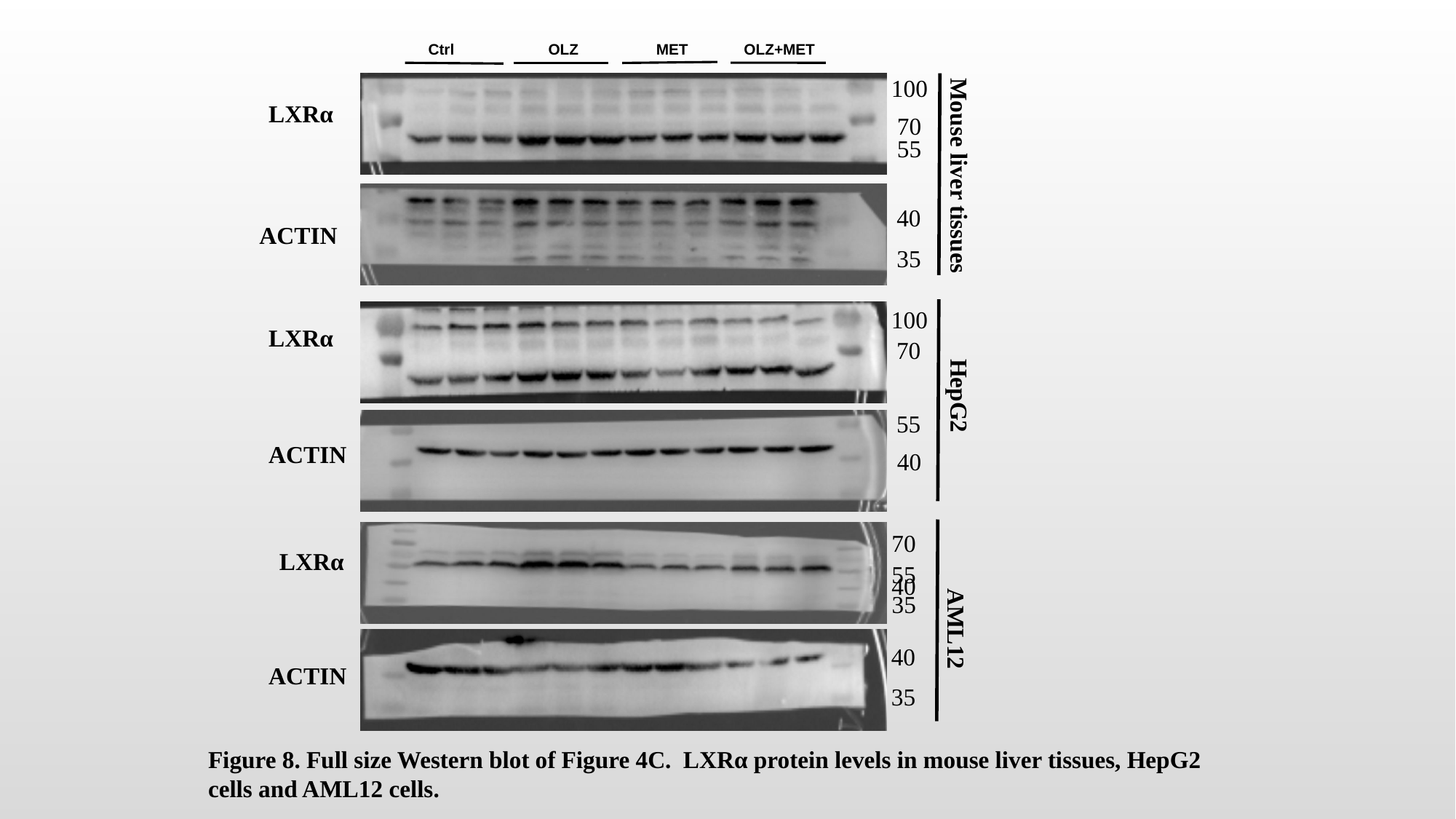

MET
Ctrl
OLZ
OLZ+MET
100
LXRα
70
55
40
ACTIN
Mouse liver tissues
35
100
LXRα
70
HepG2
55
ACTIN
40
70
LXRα
55
40
35
AML12
40
ACTIN
35
Figure 8. Full size Western blot of Figure 4C. LXRα protein levels in mouse liver tissues, HepG2 cells and AML12 cells.

## Slide 9
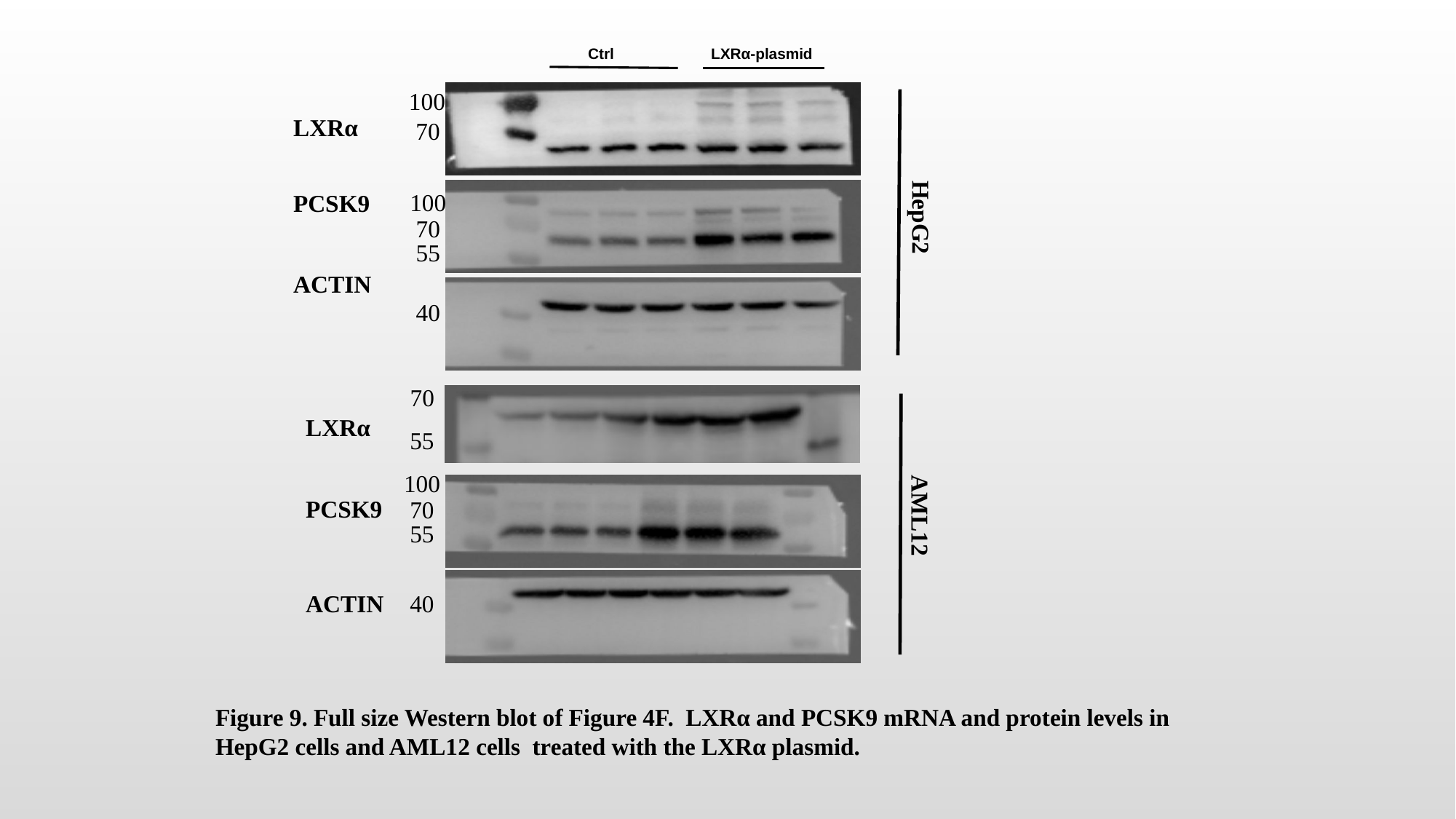

Ctrl
LXRα-plasmid
100
LXRα
70
100
PCSK9
HepG2
70
55
ACTIN
40
70
LXRα
55
100
PCSK9
70
AML12
55
40
ACTIN
Figure 9. Full size Western blot of Figure 4F. LXRα and PCSK9 mRNA and protein levels in HepG2 cells and AML12 cells treated with the LXRα plasmid.

## Slide 10
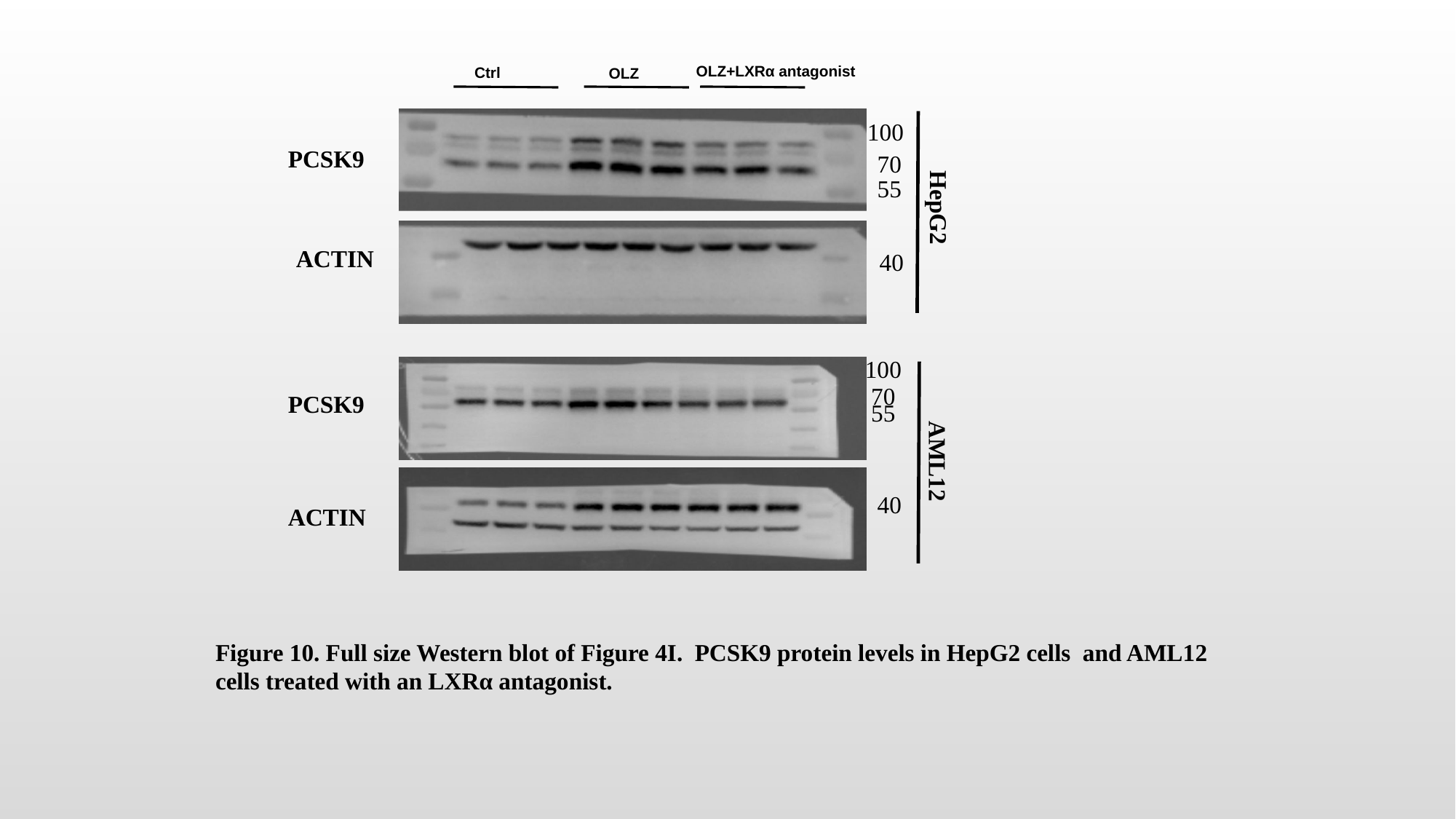

OLZ+LXRα antagonist
Ctrl
OLZ
100
PCSK9
70
55
HepG2
ACTIN
40
100
70
PCSK9
55
AML12
40
ACTIN
Figure 10. Full size Western blot of Figure 4I. PCSK9 protein levels in HepG2 cells and AML12 cells treated with an LXRα antagonist.

## Slide 11
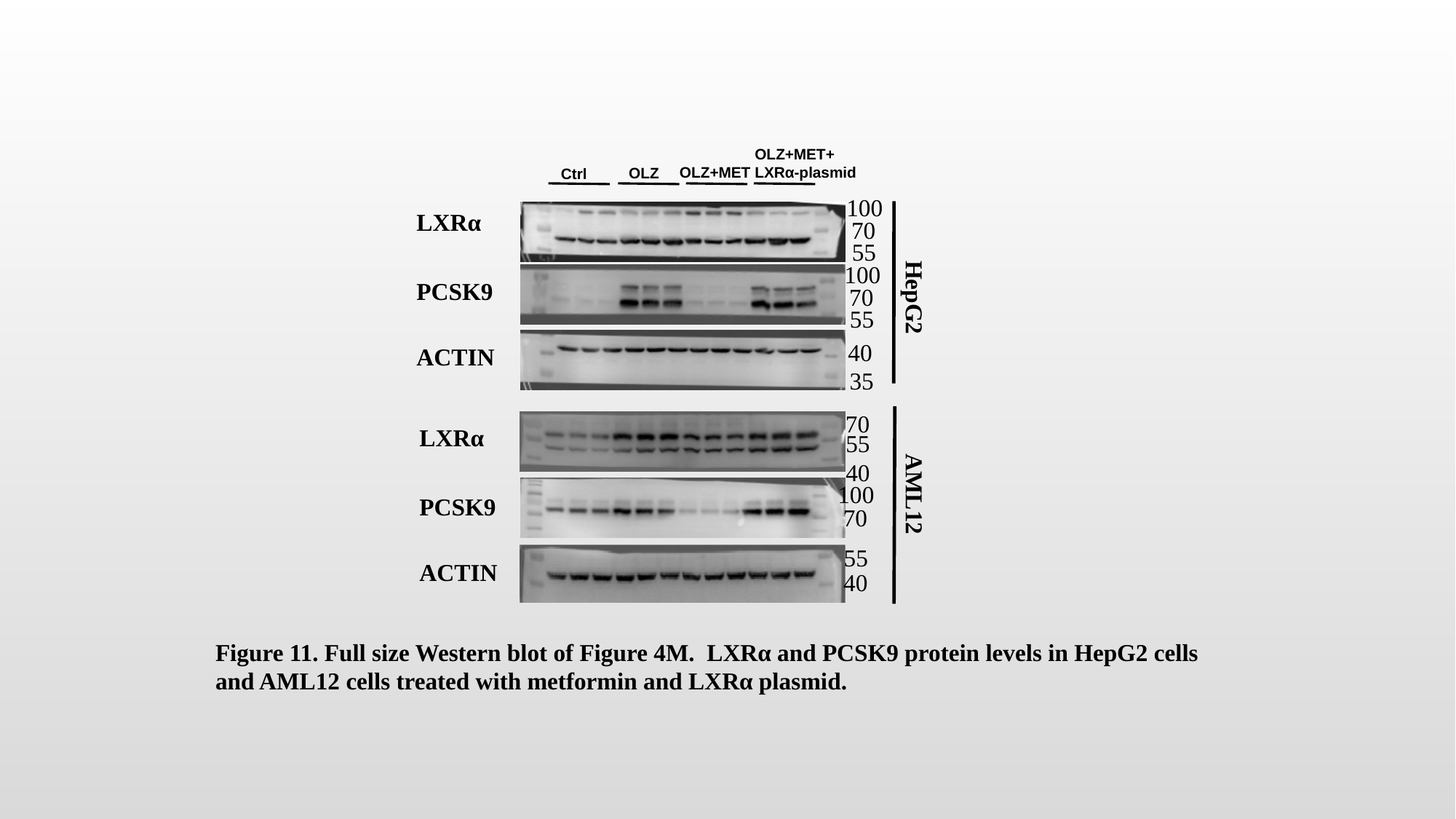

OLZ+MET+
LXRα-plasmid
OLZ+MET
OLZ
Ctrl
100
LXRα
70
55
100
PCSK9
70
HepG2
55
40
ACTIN
35
70
LXRα
55
40
100
AML12
PCSK9
70
55
ACTIN
40
Figure 11. Full size Western blot of Figure 4M. LXRα and PCSK9 protein levels in HepG2 cells and AML12 cells treated with metformin and LXRα plasmid.

## Slide 12
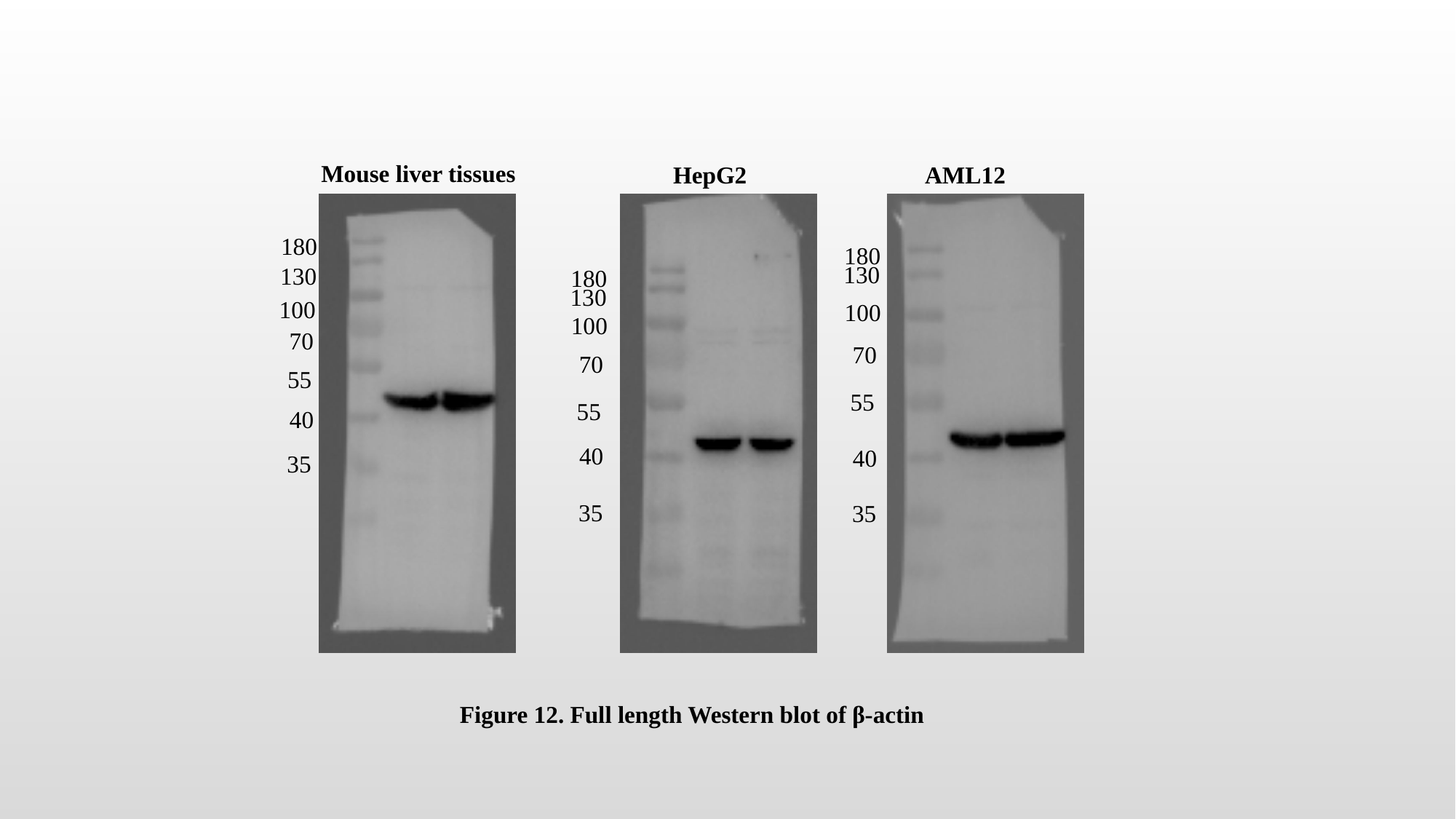

Mouse liver tissues
HepG2
AML12
180
180
130
130
180
130
100
100
100
70
70
70
55
55
55
40
40
40
35
35
35
Figure 12. Full length Western blot of β-actin

## Slide 13
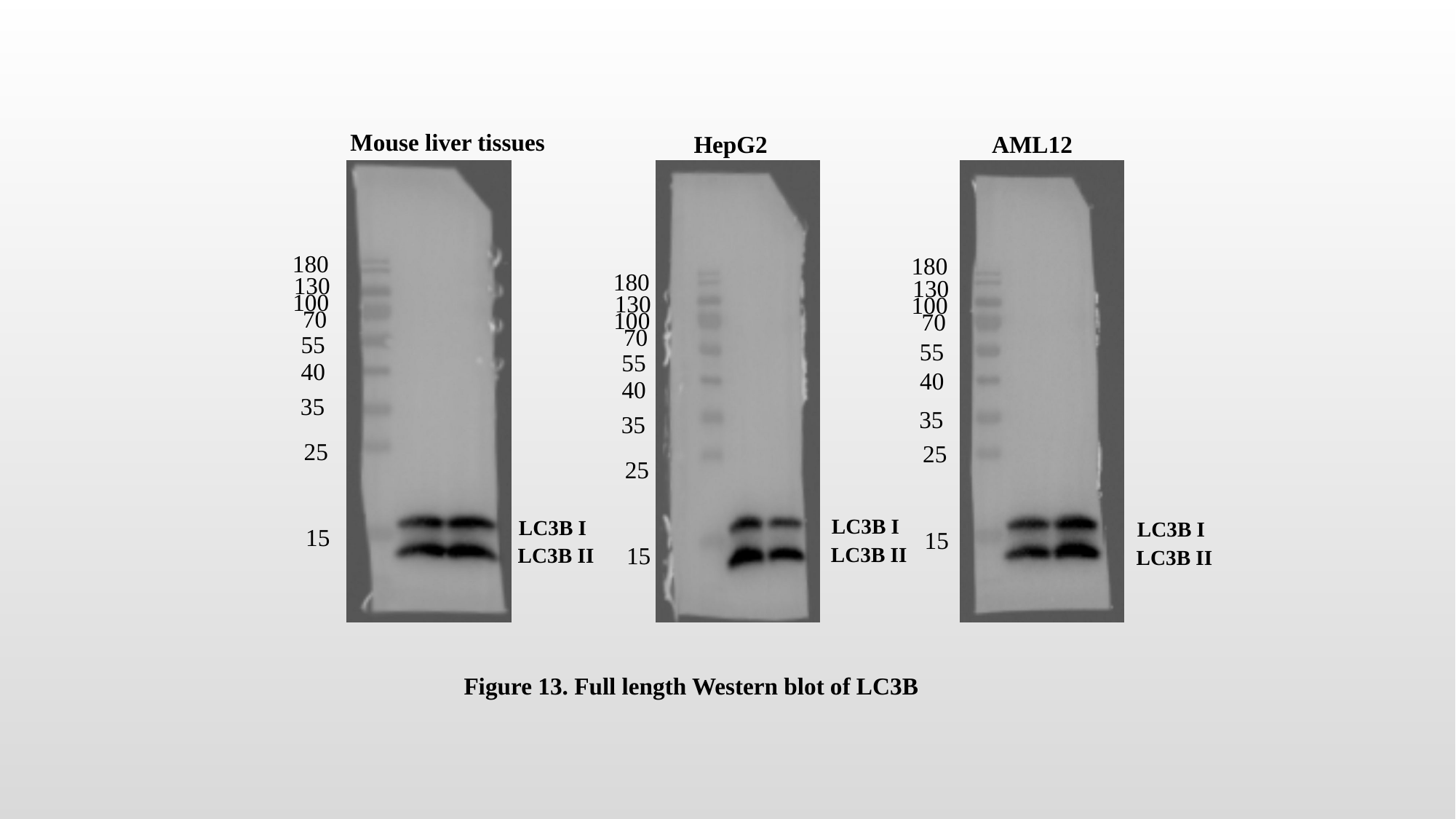

Mouse liver tissues
HepG2
AML12
180
180
180
130
130
100
130
100
70
100
70
70
55
55
55
40
40
40
35
35
35
25
25
25
LC3B I
LC3B I
LC3B I
15
15
15
LC3B II
LC3B II
LC3B II
Figure 13. Full length Western blot of LC3B

## Slide 14
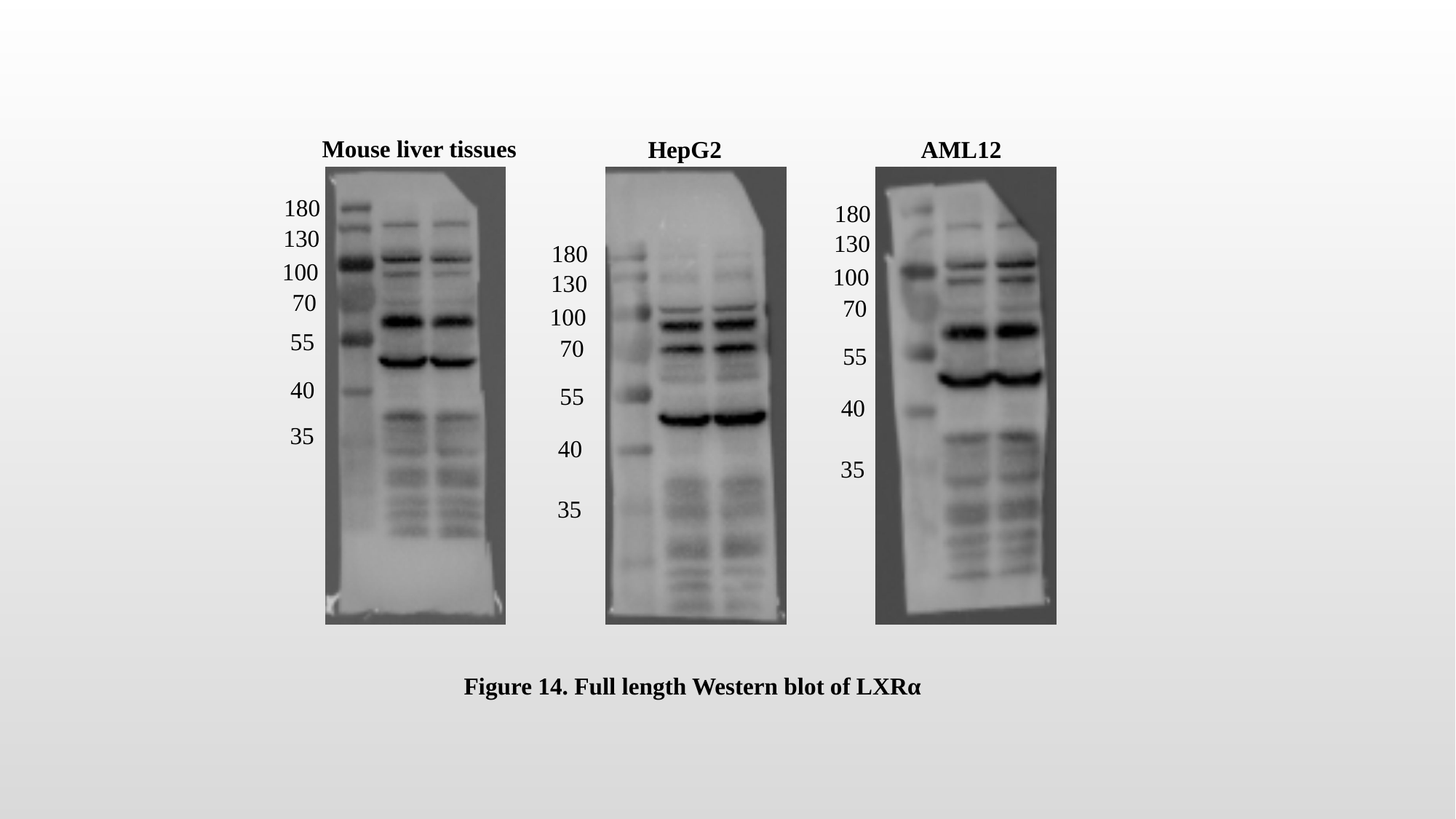

Mouse liver tissues
HepG2
AML12
180
180
130
130
180
100
100
130
70
70
100
55
70
55
40
55
40
35
40
35
35
Figure 14. Full length Western blot of LXRα

## Slide 15
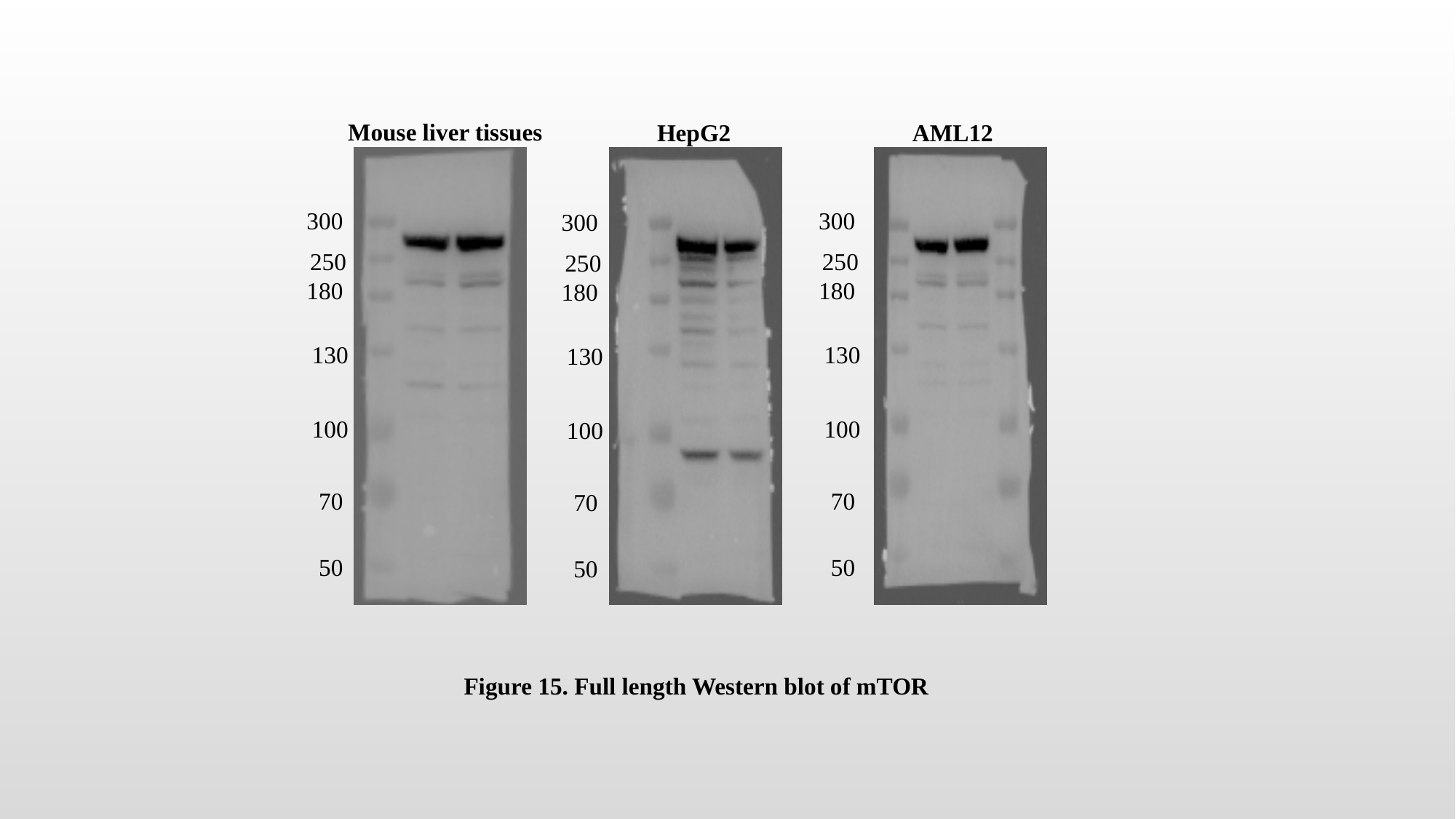

Mouse liver tissues
HepG2
AML12
300
300
300
250
250
250
180
180
180
130
130
130
100
100
100
70
70
70
50
50
50
Figure 15. Full length Western blot of mTOR

## Slide 16
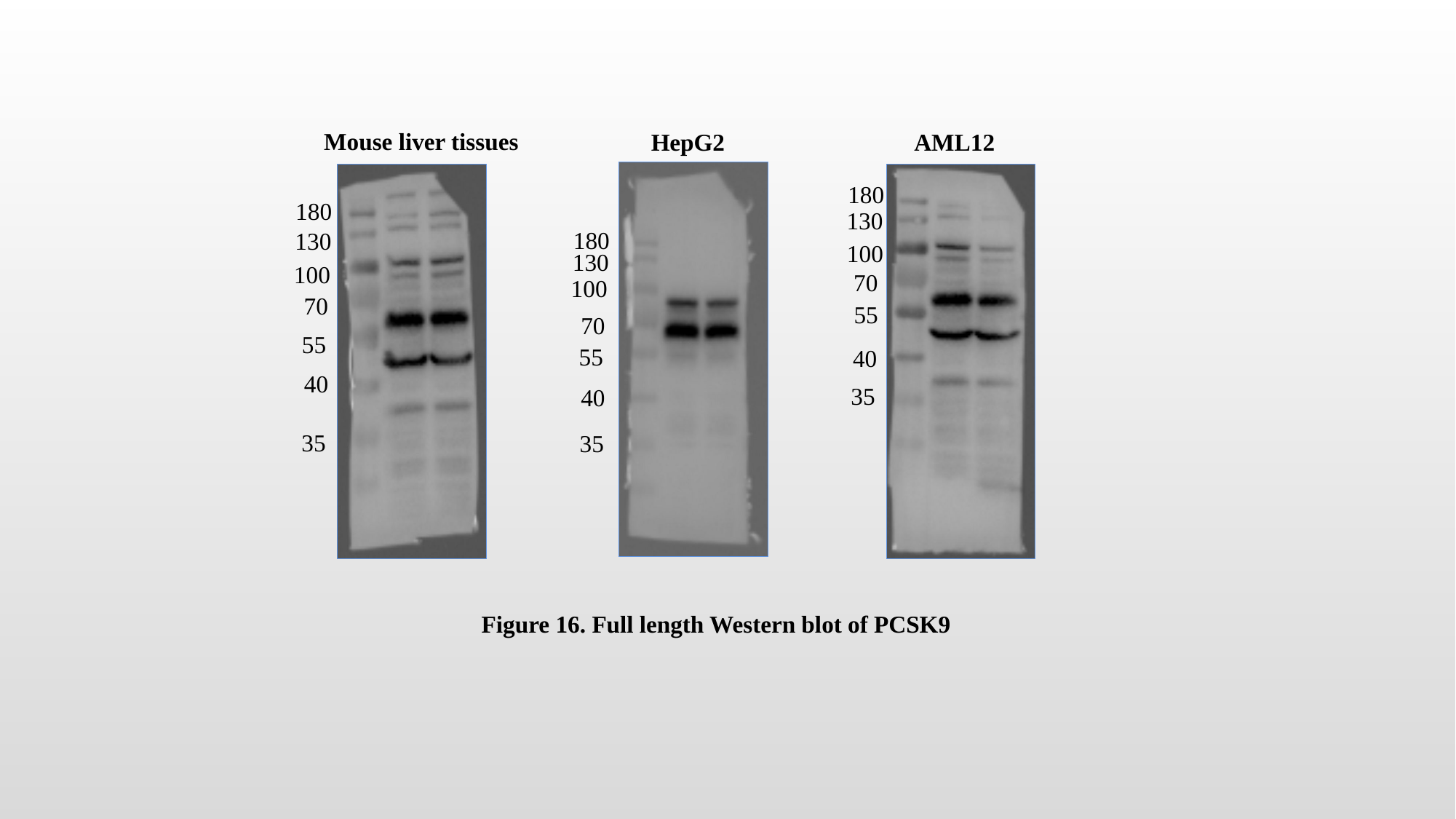

Mouse liver tissues
HepG2
AML12
180
180
130
180
130
100
130
100
70
100
70
55
70
55
55
40
40
35
40
35
35
Figure 16. Full length Western blot of PCSK9

## Slide 17
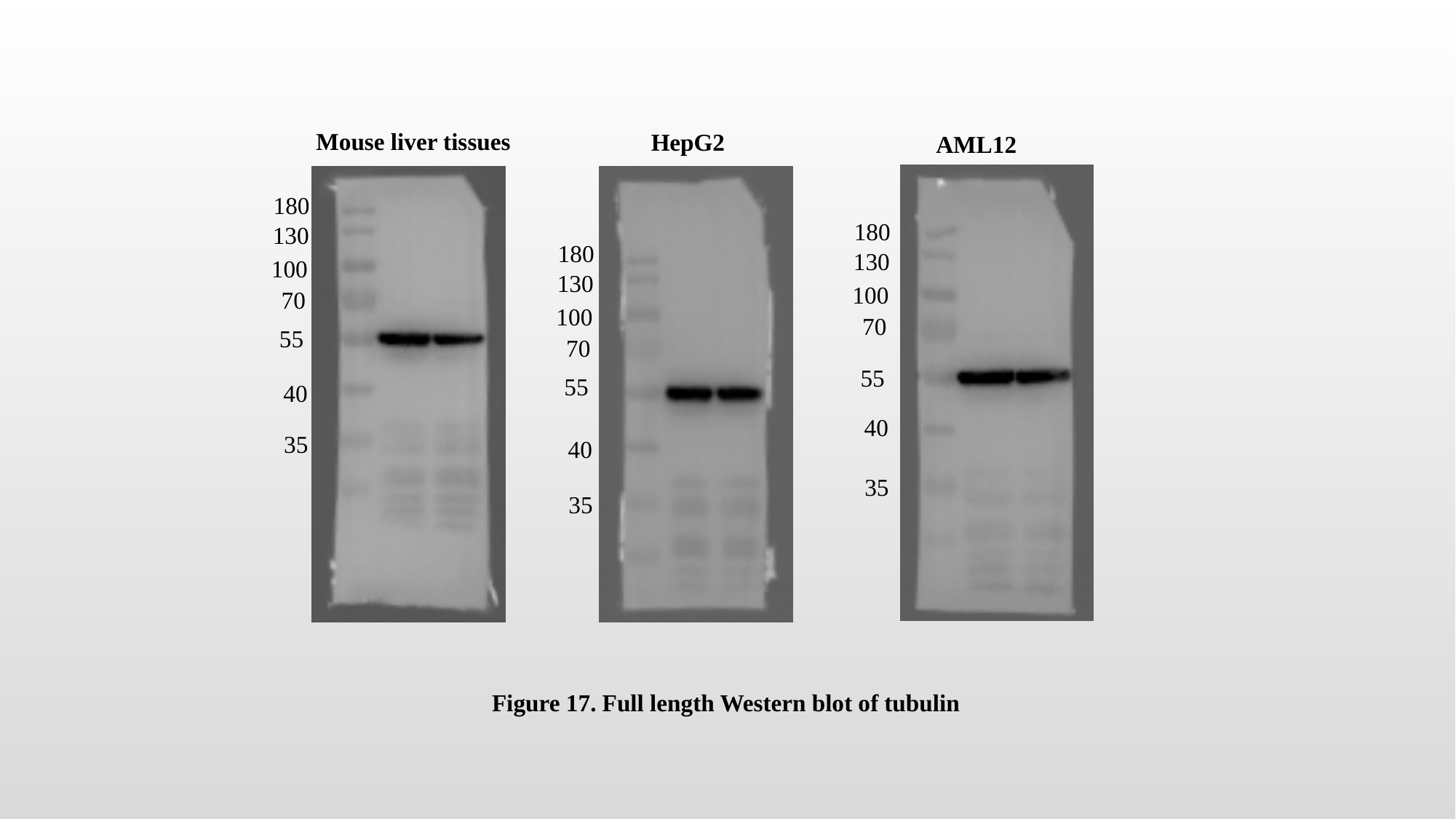

Mouse liver tissues
HepG2
AML12
180
180
130
180
130
100
130
100
70
100
70
55
70
55
55
40
40
35
40
35
35
Figure 17. Full length Western blot of tubulin

## Slide 18
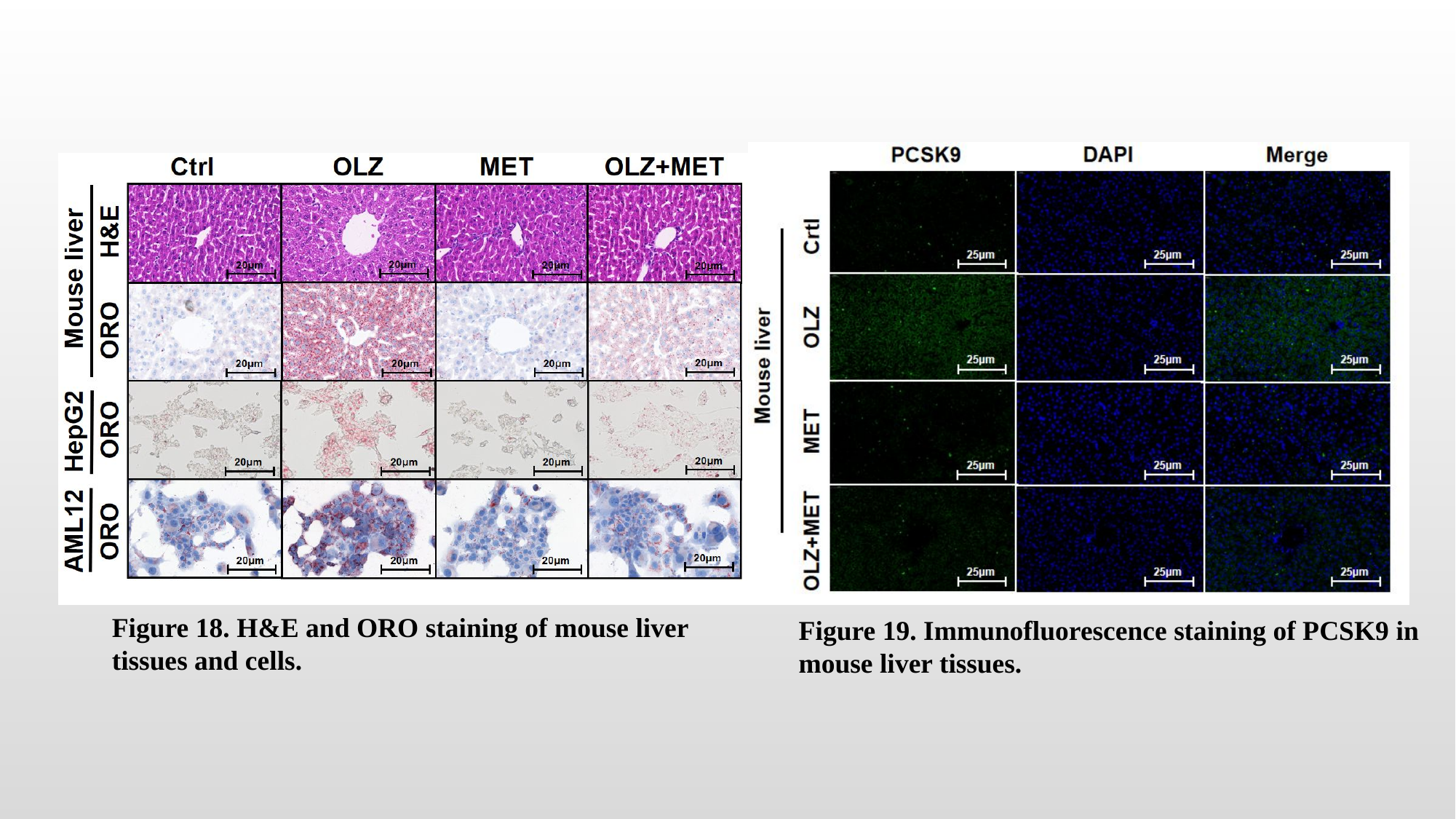

Figure 18. H&E and ORO staining of mouse liver
tissues and cells.
Figure 19. Immunofluorescence staining of PCSK9 in
mouse liver tissues.

## Slide 19
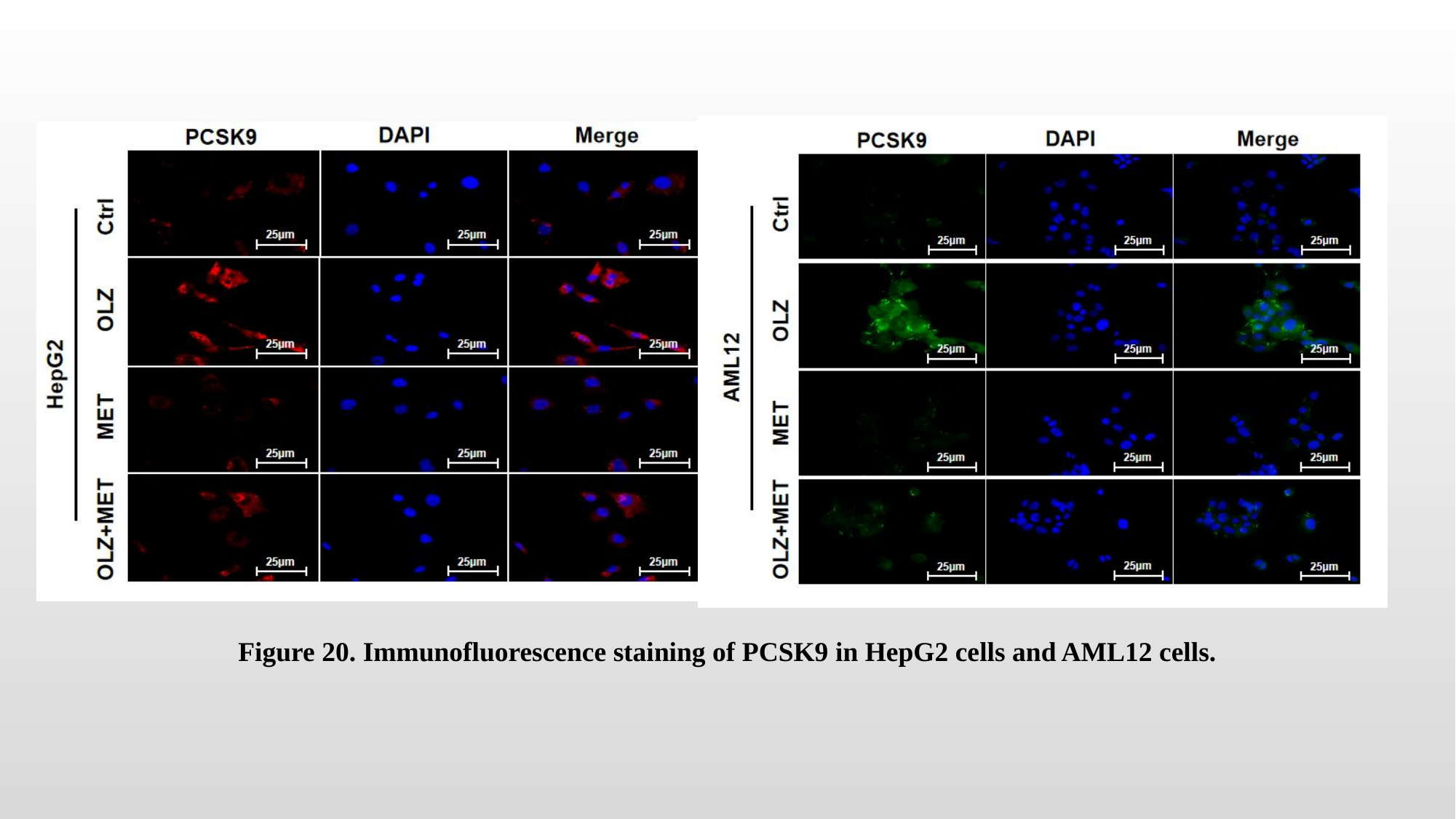

Figure 20. Immunofluorescence staining of PCSK9 in HepG2 cells and AML12 cells.

## Slide 20
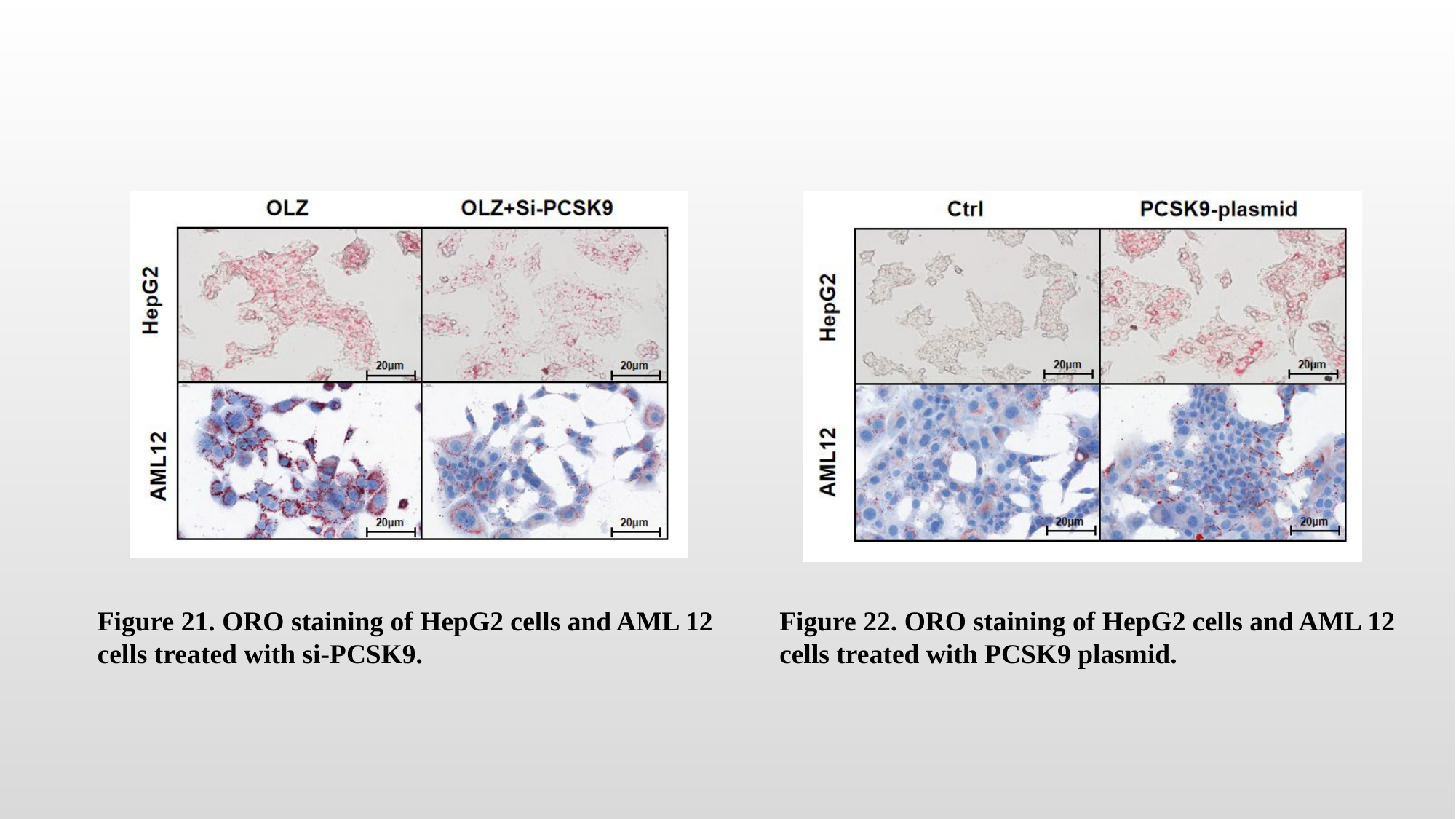

Figure 21. ORO staining of HepG2 cells and AML 12
cells treated with si-PCSK9.
Figure 22. ORO staining of HepG2 cells and AML 12
cells treated with PCSK9 plasmid.

## Slide 21
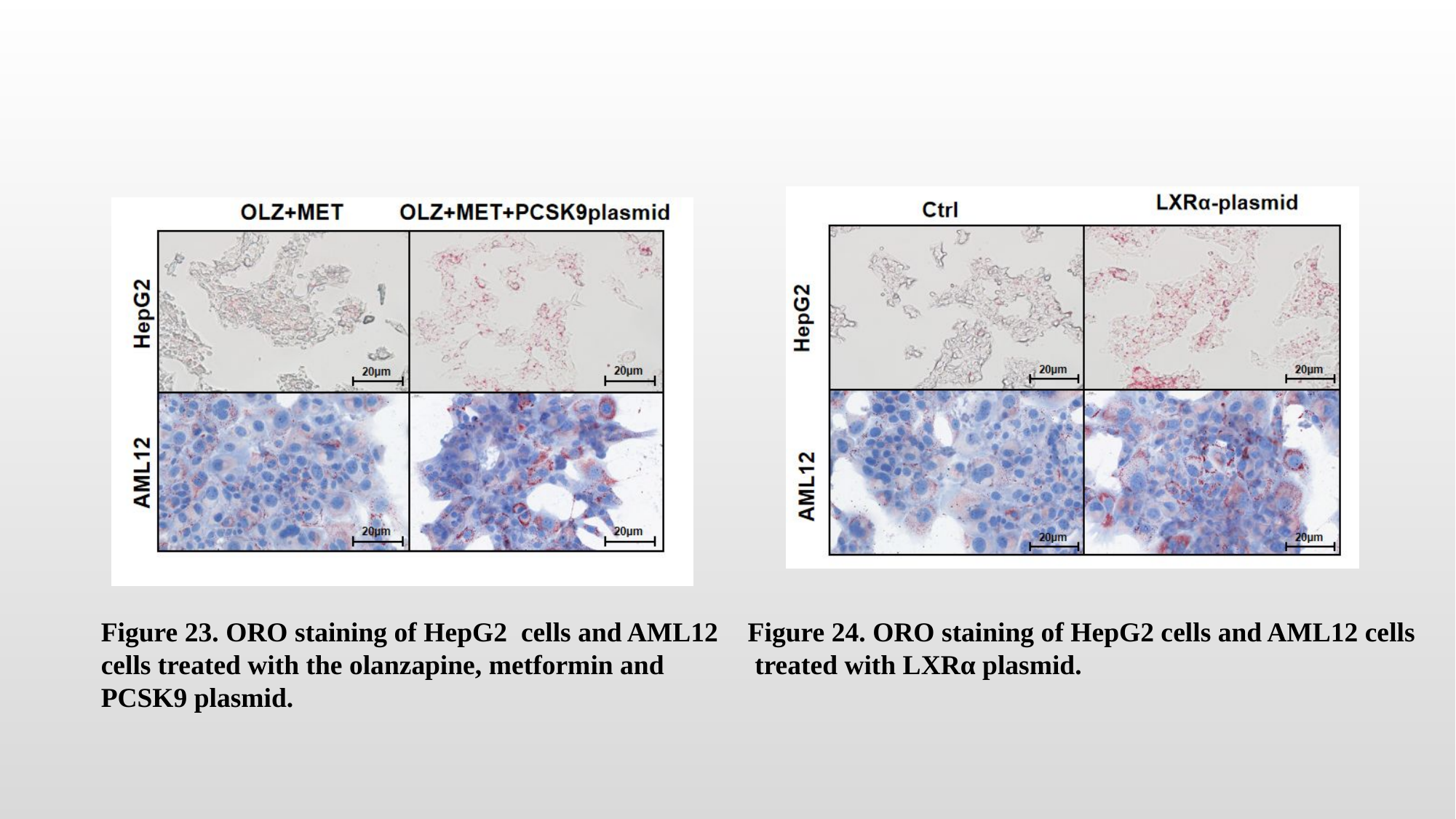

Figure 23. ORO staining of HepG2 cells and AML12
cells treated with the olanzapine, metformin and
PCSK9 plasmid.
Figure 24. ORO staining of HepG2 cells and AML12 cells
 treated with LXRα plasmid.

## Slide 22
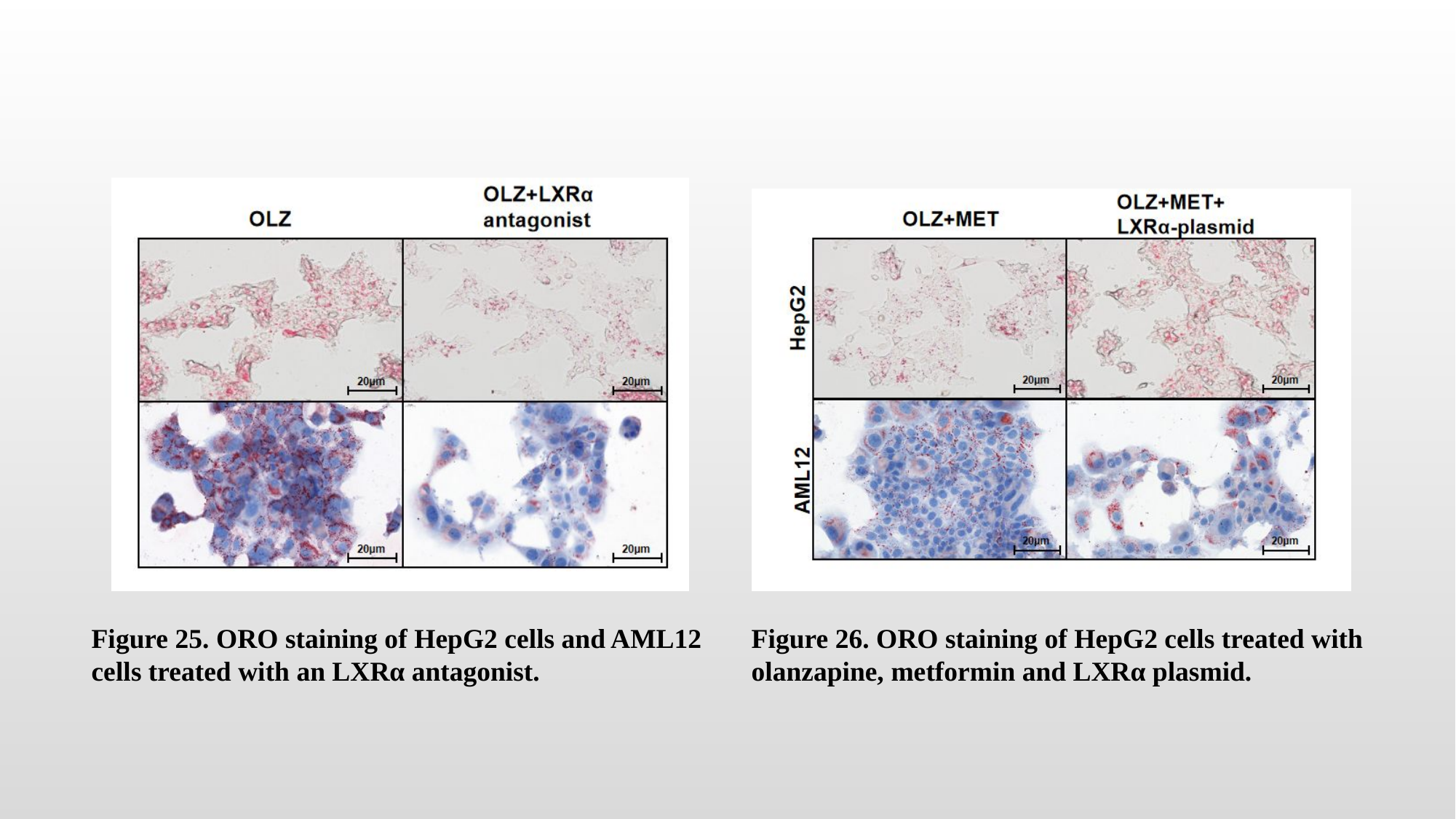

Figure 25. ORO staining of HepG2 cells and AML12
cells treated with an LXRα antagonist.
Figure 26. ORO staining of HepG2 cells treated with
olanzapine, metformin and LXRα plasmid.
